# A novel fatty acid mimetic with pan-PPAR partial agonist activity inhibits diet-induced obesity and metabolic dysfunction-associated steatotic liver disease

**DOI:** 10.1016/j.molmet.2024.101958

**Published:** 2024-05-17

**Authors:** Cigdem Sahin, Jenna-Rose Melanson, Florian Le Billan, Lilia Magomedova, Thais A.M. Ferreira, Andressa S. Oliveira, Evan Pollock-Tahari, Michael F. Saikali, Sarah B. Cash, Minna Woo, Luiz A.S. Romeiro, Carolyn L. Cummins

**Affiliations:** 1 Department of Pharmaceutical Sciences, Leslie Dan Faculty of Pharmacy, University of Toronto, Toronto, ON M5S 3M2, Canada; 2 Department of Pharmacy, Faculty of Health Sciences, University of Brasilia, Brasilia, DF 71910-900, Brazil; 3 Toronto General Hospital Research Institute, University Health Network, Toronto, ON, M5G 2C4, Canada; 4 Banting and Best Diabetes Centre, Toronto, ON, M5G 2C4, Canada

**Keywords:** FGF21, Adipose, LDT409, Diabetes, NAFLD, MASLD

## Abstract

**Objective:**

The prevalence of metabolic diseases is increasing globally at an alarming rate; thus, it is essential that effective, accessible, low-cost therapeutics are developed. Peroxisome proliferator-activated receptors (PPARs) are transcription factors that tightly regulate glucose homeostasis and lipid metabolism and are important drug targets for the treatment of type 2 diabetes and dyslipidemia. We previously identified LDT409, a fatty acid-like compound derived from cashew nut shell liquid, as a novel pan-active PPARα/γ/δ compound. Herein, we aimed to assess the efficacy of LDT409 *in vivo* and investigate the molecular mechanisms governing the actions of the fatty acid mimetic LDT409 in diet-induced obese mice.

**Methods:**

C57Bl/6 mice (6–11-month-old) were fed a chow or high fat diet (HFD) for 4 weeks; mice thereafter received once daily intraperitoneal injections of vehicle, 10 mg/kg Rosiglitazone, 40 mg/kg WY14643, or 40 mg/kg LDT409 for 18 days while continuing the HFD. During treatments, body weight, food intake, glucose and insulin tolerance, energy expenditure, and intestinal lipid absorption were measured. On day 18 of treatment, tissues and plasma were collected for histological, molecular, and biochemical analysis.

**Results:**

We found that treatment with LDT409 was effective at reversing HFD-induced obesity and associated metabolic abnormalities in mice. LDT409 lowered food intake and hyperlipidemia, while improving insulin tolerance. Despite being a substrate of both PPARα and PPARγ, LDT409 was crucial for promoting hepatic fatty acid oxidation and reducing hepatic steatosis in HFD-fed mice. We also highlighted a role for LDT409 in white and brown adipocytes *in vitro* and *in vivo* where it decreased fat accumulation, increased lipolysis, induced browning of WAT, and upregulated thermogenic gene *Ucp1*. Remarkably, LDT409 reversed HFD-induced weight gain back to chow-fed control levels. We determined that the LDT409-induced weight-loss was associated with a combination of increased energy expenditure (detectable before weight loss was apparent), decreased food intake, increased systemic fat utilization, and increased fecal lipid excretion in HFD-fed mice.

**Conclusions:**

Collectively, LDT409 represents a fatty acid mimetic that generates a uniquely favorable metabolic response for the treatment of multiple abnormalities including obesity, dyslipidemia, metabolic dysfunction-associated steatotic liver disease, and diabetes. LDT409 is derived from a highly abundant natural product-based starting material and its development could be pursued as a therapeutic solution to the global metabolic health crisis.

## Highlights

• LDT409 promotes weight loss in HFD-fed mice, not lean mice.
• Decreased food intake and increased fat utilization drive LDT409-induced weight loss.
• LDT409 improves glucose homeostasis and plasma lipids in HFD-fed mice.
• Hepatic fat accumulation is reduced with LDT409 treatment.
• LDT409 generates a unique metabolic profile compared to full PPAR agonists.

## Graphical abstract

**Figure:**
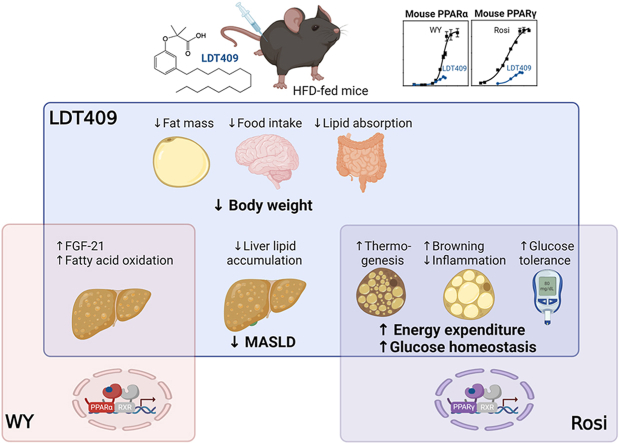
LDT409 is a pan-PPAR partial agonist that promotes an array of metabolic improvements in diet-induced obese mice.

## Abbreviations

Acaca: acetyl-CoA carboxylase alpha
Alpl: alkaline phosphatase biomineralization associated
BAT: brown adipose tissue
Cd36: fatty acid translocase
Cd68: cluster of differentiation 68
Chrebp: carbohydrate-responsive element-binding protein
Cidea: cell death-inducing DFFA-like effector a
Cidec: cell death-inducing DFFA-like factor c
Ckb: creatine kinase b
Cox7a: cytochrome c oxidase subunit 7a
Cox8b: cytochrome c oxidase subunit 8b
Dgat1/2: diglyceride acyltransferase 1 or 2
DIO: diet-induced obesity
Elovl3: fatty acid elongase 3
Fabp1: fatty acid-binding protein 1
Fabp4: fatty acid binding protein 4
Fasn: fatty acid synthase
FFA: free fatty acid
Fgf21: fibroblast growth factor 21
DEXA: dual energy x-ray absorptiometry
Gpr3: G-protein coupled receptor 3
GTT: glucose tolerance test
H&E: hematoxylin and eosin
HFD: high-fat diet
Il-1β: interleukin 1 beta
ITT: insulin tolerance test
Lpl: lipoprotein lipase
MASLD: metabolic dysfunction-associated steatotic liver disease
Mcp1: monocyte chemoattract protein 1
NAFLD: non-alcoholic fatty liver disease
NEFA: non-esterified fatty acid
OEA: oleoylethanolamide
Pck1: phosphoenolpyruvate carboxykinase
Pdk4: pyruvate dehydrogenase kinase 4
Prdm16: PR/SET domain 16
PPAR: peroxisome proliferator-activated receptor
Ppargc1α: peroxisome proliferator-activated receptor-gamma coactivator alpha 1
RER: respiratory exchange ratio
Srebp1c: sterol regulatory element-binding protein 1c
Scd-1: stearoyl-CoA desaturase-1
Tnfα: tumor necrosis factor alpha
TZD: thiazolidinedione
Ucp1: uncoupling protein 1
WAT: white adipose tissue

## 1 Introduction

Over the last three decades, there has been a progressive increase in the global prevalence of obesity and diabetes [[1], [2], [3], [4]]. The pathophysiology of obesity is characterized by the development of dysfunctional adipose tissue with increased visceral fat mass, enlarged adipocyte size, increased immune cell infiltration, altered secretion of adipokines, and increased circulating free fatty acids (FFAs) [1,2,5,6]. Obesity is a significant risk factor for dyslipidemia, insulin resistance and metabolic dysfunction-associated steatotic liver disease (MASLD) that strongly predispose individuals to develop type 2 diabetes [1,2,7,8].

Adipose tissue can be classified based on its role in overall systemic energy homeostasis [5,9,10]. Healthy white adipose tissue (WAT) efficiently stores excess energy as triglycerides and secretes adipokines such as adiponectin that improve insulin sensitivity. In contrast, brown adipose tissue (BAT) and beige adipocytes use their abundant mitochondria to oxidize substrates (e.g., fat) and produce heat through mitochondrial uncoupling protein-1 (UCP1) [10,11] and other futile cycles (calcium cycling, creatine cycling and lipolysis/re-esterification cycles) that help to maintain core body temperature in response to environmental stimuli such as cold exposure [9,[12], [13], [14], [15], [16], [17], [18], [19]].

The PPARs (PPARα, PPARδ, and PPARγ) are members of the nuclear receptor superfamily that are endogenously stimulated by fatty acids, fatty acid-derivatives, and eicosanoids [20]. Each PPAR isoform exhibits unique functions depending on the tissue in which it is expressed and the presence of different endogenous ligands. PPARα upregulates fatty acid uptake and fatty acid utilization in the liver, while it increases fatty acid oxidation and thermogenesis in BAT [[21], [22], [23]]. PPARδ enhances glucose uptake, fatty acid oxidation, and oxidative capacity in skeletal muscle, which improves insulin sensitivity and energy utilization [24,25]. PPARγ is a key regulator of adipogenesis in WAT and BAT, where it activates thermogenesis and promotes adipokine secretion that contributes to improving systemic insulin sensitivity [22,26]. There are drugs currently on the market that target two of these receptors (fibrates: PPARα agonists; thiazolidinediones: PPARγ agonists) but are not without drawbacks. Fibrates are prescribed for the treatment of hypertriglyceridemia, but show increased creatinine and are of limited use in patients chronic kidney disease [27,28]. The thiazolidinediones (TZDs) are drugs that target PPARγ and are highly effective for the treatment of insulin resistance in patients with type 2 diabetes [26,29]. However, TZDs cause serious side effects (e.g., weight gain, edema, bone fracture, and heart failure) that have severely limited their clinical utility [26,[29], [30], [31], [32]]. The side-effects of TZDs have been attributed to their high affinity and high potency for PPARγ [31]. Desirable features of new generation PPAR agonist would be to mimic the endogenous ligands by having balanced affinities for PPARα and PPARγ, and partial agonism for PPARγ. Previous studies show that the endogenous fatty acid derivative and ligand for PPARα, oleoylethanolamide (OEA), protects against high-fat diet (HFD)-induced fatty liver, and stimulates weight loss in rodents [[33], [34], [35]]. Activation of PPARγ with the endogenous ligand decanoic acid shows improvement of insulin sensitivity without inducing weight-gain [36]. Given the myriad beneficial effects of endogenous PPAR activation on metabolism, a small molecule that could engage the receptors in a way that mimicked the endogenous ligands could provide enhanced metabolic outcomes.

We identified LDT409 as a fatty acid-mimetic due to its structural features of having a 15-carbon long tail and phenolic group with a carboxylic acid [37]. LDT409 is easily synthesized from an abundant natural waste by-product of the cashew nut shell industry which is widely available in low- and middle-income countries [38]. LDT409 is a single molecule that induces partial agonism against human PPARα (EC_50_ 0.5 μM) and PPARγ (EC_50_ 0.9 μM) with weak binding affinity to PPARδ (EC_50_ 30 μM) [37]. Herein, we investigated the impact of this fatty acid mimetic in a mouse model of diet-induced obesity and compared its effects to classic full agonists of PPARα (WY14643; WY) and PPARγ (Rosiglitazone; Rosi). Our results demonstrate that LDT409 represents a fatty acid mimic that generates a uniquely favorable metabolic response for the treatment of multiple sequelae of metabolic disease including obesity, type 2 diabetes, dyslipidemia and MASLD.

## 2 Material and methods

### 2.1 Reagents

High glucose DMEM D5796, DMEM/Ham's, penicillin/streptomycin, 0.25% trypsin, lipopolysaccharides (LPS), insulin, isoproterenol, and triiodothyronine, were purchased from Sigma–Aldrich (St. Louis, MO). Fetal bovine serum was purchased from Invitrogen (Carlsbad, CA). Fetal calf serum was purchased from ThermoFisher Scientific (Waltham, MA). Glutamine and HEPES were purchased from Life Technologies (Carlsbad, CA). The Agilent Seahorse XF Cell Mito Stress test kit, including oligomycin, FCCP, antimycin, rotenone was bought from Agilent (Santa Clara, CA). Rosiglitazone and WY14643 were purchased from Cayman Chemical Company (Ann Arbor, MI, USA). LDT409 was synthesized as described previously [37].

### 2.2 Animal studies

Wild-type (WT) mice were bred and housed at the Division of Comparative Medicine within a standard temperature and light-controlled environment. All mice used in the experiments were C57Bl/6 age-matched from 6 to 11 months. All procedures were approved by the Faculties of Medicine and Pharmacy Animal Care Committee (FMPACC) at the University of Toronto (Toronto, ON) or the University Health Network (Toronto, ON). Mice were fed a standard rodent chow diet (Envigo 2016S rodent diet, Harlan Teklad, Mississauga, ON, Canada).

For diet-induced obesity (DIO) metabolic phenotyping, mice were fed a high-fat diet (HFD) containing 42% kcal from fat and 0.2% cholesterol by weight (Envigo TD.88137, Harlan Teklad, Mississauga, ON, Canada) for 6.5 weeks. Experiments were performed with male mice unless otherwise stated. After 4 weeks on HFD, mice were then randomly divided into 3 groups to receive intraperitoneal injection of vehicle, Rosi (10 mg/kg), WY (40 mg/kg) or LDT409 (40 mg/kg) once a day for 18 days. Compounds were formulated in 5% DMSO and 5% Tween-80 with 0.9% NaCl. Rosi and WY were made fresh every 2 days to prevent precipitation, while LDT409 was prepared weekly. Control groups for chow-fed and HFD-fed mice were injected with vehicle consisting of 5% DMSO and 5% Tween-80 with 0.9% NaCl. In a separate cohort, chow-fed male WT mice (7–8 months) were divided into two groups to receive intraperitoneal injection of vehicle or LDT409 (40 mg/kg) once a day for 18 days. Body weight was measured weekly and food intake was determined biweekly by measuring remaining food in the hopper and inspecting cages bottom for food spillage. Mice were individually housed during the study. On the last day of treatment, drugs were administered at 8 a.m. and animals were sacrificed by decapitation at 10 a.m. Tissue samples were harvested, weighed, snap frozen in liquid nitrogen, and stored at −80 °C until processing. The right femur from each mouse was isolated and cleaned, and then femur length was measured using a digital caliper (Neiko Tools, Taiwan). Trunk blood was collected in tubes containing 5 μL of 0.5 M EDTA and plasma was separated by centrifugation at 500×*g*, 4 °C for 20 min.

### 2.3 Pair-feeding study

WT male C57Bl/6 mice (6–7 months old) were fed a chow diet or HFD diet. After 4 weeks, the HFD-fed mice were randomized to receive daily intraperitoneal injections of vehicle (5% DMSO and 5% Tween-80 in 0.9% NaCl) or 40 mg/kg LDT409 for 18 days while continuing the HFD. HFD-fed pair-fed and chow-fed mice received only vehicle injections. The pair-fed group was provided the same amount of food consumed by LDT409-treated mice, which was measured daily and adjusted accordingly for the pair-fed mice.

### 2.4 Indirect calorimetry

WT male C57Bl/6 mice (10–11 months old) were fed a chow diet or HFD diet for 4 weeks and then transferred to the Animal Research Facility at the University Health Network. Mice were acclimatized for 3 days before starting ligand treatment. Indirect calorimetry was performed for each mouse, single-housed at room temperature in Oxymax Comprehensive Laboratory Animal Monitoring System (CLAMS, Columbus Instruments, Columbus, OH) metabolic cages. Mice were housed in the CLAMS cage systems for 72 h between days 3–6 of treatment, and days 14–17 of treatment with *ad libitum* access to food and water. Volume of O_2_ consumed and CO_2_ produced, physical activity, food intake, and water consumption were recorded by the Oxymax indirect calorimeter system (Columbus Instruments). For studies performed at thermoneutrality (30 °C), 16 Promethion cages (Sable Systems International, Las Vegas, NV) located in a temperature-controlled cabinet at the University of Toronto Department of Comparative Medicine were used. To begin, WT male C57Bl/6 mice (8-months old) were fed HFD for 4 weeks while housed at room temperature. Mice were then transferred to the Promethion metabolic cages, singly housed with free access to HFD and water, while the chamber temperature was progressively increased from room temperature to thermoneutrality. The following day, vehicle or 40 mg/kg LDT409 intraperitoneal injections were initiated. Whole body metabolic rate (volume of O_2_ consumption and CO_2_ production) energy expenditure and balance, respiratory exchange ratio, activity, food intake, and water consumption were continuously measured over the 14-day period.

### 2.5 Lipid absorption

Stools were collected from single-housed mice over 72 h. The Folch method was used to extract lipid from feces using chloroform/methanol [39,40]. Lipid content of the diet and stools was assessed gravimetrically, and intestinal lipid absorption rate was calculated [41]. Fecal triglyceride and cholesterol content was also measured from dried aliquots of standards and samples using the colorimetric Infinity Triglyceride and Cholesterol reagents (ThermoFisher Scientific).

### 2.6 Measurement of fat content by DEXA scan

Body composition was measured in mice using dual energy X-ray absorptiometry (DEXA, Bruker In-Vivo Xtreme, Billerica, MA). DEXA scan was completed according to manufacturer's instructions. Briefly, mice were anesthetized with 2–3% isoflurane-oxygen and kept in a prone position until DEXA images were obtained (5 min). X-ray images were taken at different energy levels to image hard tissues (lean and bone) vs total tissues (lean, bone, and fat) by using 0.8 mm AI and 0.0 mm AI filters, respectively. The images were corrected for illumination and converted to density units by the instrument software. Based on the attenuation of two energy levels, the system provides quantitative data on the hard tissue content and total tissue mass content within the region of interest (ROI). The head was not included in the ROI. For the calculation of total fat percent, the hard tissue ROI was subtracted from all tissue mass ROI, divided by the total tissue ROI, and then multiplied by 100.

### 2.7 Glucose and insulin tolerance tests

On day 12 of treatment, a glucose tolerance test (GTT) was performed on mice fasted for 16 h with *ad libitum* access to water. Following an initial blood glucose measurement, 1 g d-glucose/kg of body weight was intraperitoneally delivered, and blood glucose levels were monitored from the tail-tip using a glucometer (Abbott, Chicago, IL) at the indicated times (15, 30, 60, and 90 min). For insulin tolerance test (ITT), mice were fasted for 4 h and 1U insulin/kg body weight was intraperitoneally injected on day 16 of treatment. Tail-vein blood glucose was measured using a glucometer (Abbott) in the basal state and at 15, 30, 45, 60, and 90 min following insulin administration.

### 2.8 Luciferase assays

Human embryonic kidney (HEK293) cells were cultured in Dulbecco's modified Eagle's medium supplemented with 10% fetal bovine serum. Cell transfection was performed in media containing 10% charcoal-stripped fetal bovine serum using calcium phosphate in 96-well plates. The total DNA transfected included 50 ng UAS-luciferase reporter, 20 ng β-galactosidase, 15 ng nuclear receptor (GAL4-mPPARα, GAL4-mPPARγ, or GAL4-mPPARδ) and pGEM filler plasmid. Six hours post-transfection, cells were treated with ligands and then harvested 16 h after ligand addition. Luciferase was measured and normalized to β-galactosidase activity to control for transfection efficiency.

### 2.9 RAW264.7 cell culture

Mouse RAW264.7 macrophage-like cells were maintained in DMEM D5796 medium, containing 10% fetal bovine serum and 1X penicillin/streptomycin at 37 °C, 5% CO_2_. Cells were treated with vehicle or 25 μM LDT409 for 24 h. Afterwards, cells were co-treated with 10 ng/mL LPS ± the indicated ligands for an additional 6 h and then cells were harvested for RNA extraction.

### 2.10 T37i cell culture and differentiation

T37i brown pre-adipocyte cells were obtained from Dr. Marc Lombès (Paris-Saclay University, France). T37i cells were cultured in DMEM/Ham's medium supplemented with 10% fetal calf serum, 1X penicillin/streptomycin, 2 mM glutamine, and 20 mM HEPES in 100 mm tissue culture plates and incubated at 37 °C in a humid atmosphere maintained with 5% CO_2_. Under active use, T37i cells were routinely passaged twice a week at a 1:10 ratio when they reached ∼90% confluence. For differentiation into brown adipocytes, T37i cells were seeded into 6-well plates and then treated with 20 nM insulin and 2 nM triiodothyronine (T_3_) for 8 days [42]. On day eight, differentiated T37i brown adipocytes were treated with vehicle (DMSO) or 25 μM LDT409 for 16 h. Cells were harvested for RNA extraction as well as media collected for *in vitro* lipolysis.

### 2.11 Oxygen consumption rate assay

On day eight, differentiated T37i brown adipocytes were seeded into 96-well Seahorse plate at 25,000 cells/well for 5 h and then treated with vehicle (DMSO) or 25 μM LDT409 for 16 h. The following day, the media was replaced 1 h before the measurement with Seahorse XF RPMI medium supplemented with 0.5 mM pyruvate, 17.5 mM glucose, and 2 mM glutamine (Agilent, Santa Clara, CA). The oxygen consumption rate (OCR) was measured using a Seahorse XFe96 analyzer (Agilent) with a Seahorse XF Cell Mito Stress Test kit, including oligomycin, FCCP, antimycin, rotenone (Agilent), which were titrated to ensure maximal effect for measurement of mitochondrial oxidation. After measuring basal oxygen consumption in the cells, 2 μM oligomycin (leak respiration), 0.5 μM FCCP (maximal respiratory capacity), and 0.5 μM antimycin A/rotenone (non-mitochondrial respiration) were added into the wells and oxygen consumption was measured under each condition during the assay. Data are presented as the average OCR that was normalized to cell numbers counted with BioTek cell counter (Agilent) immediately after Seahorse assay using Hoechst fluorescent dye (Sigma–Aldrich, St. Louis, MO).

### 2.12 Plasma analyses

Trunk blood was collected in EDTA-covered tubes on ice, centrifuged at 500×*g*, 4 °C for 20 min, and then plasma was stored at −80 °C. Plasma cholesterol (ThermoFisher Scientific), triglycerides (Wako, Richmond, VA), glucose (Wako), non-esterified fatty acids (NEFA; Wako), glycerol (Sigma–Aldrich), and β-hydroxybutyrate (Sigma–Aldrich) were measured by colorimetric assays. Insulin was measured by radioimmunoassay (RIA, Millipore, Bedford, MA). Fibroblast growth factor 21 (FGF21) was determined using an ELISA kit (BioVendor, Asheville, NC).

### 2.13 RNA isolation, cDNA synthesis, and real-time QPCR analysis

Total RNA was extracted from cells and tissues using RNA STAT-60 (Tel-Test Inc, Friendswood, TX) or TRIzol® Reagent (ThermoFisher Scientific). A total of 2 μg RNA was treated with Dnase I and reverse transcribed into cDNA with random hexamer primers using the High-Capacity Reverse Transcription System (Applied Biosystems (ABI), Burlington, ON, Canada). Real-time quantitative PCR (QPCR) reactions were performed on an ABI 7900 in 384-well plates containing 12.5 ng cDNA, 150 nM each of forward and reverse primers, and 5 μL 2X SYBR Green PCR Master Mix (ABI, Burlington, ON, Canada) in a total volume of 10 μL. Relative mRNA levels were calculated using the comparative Ct method normalized to cyclophilin or 36B4 mRNA. Primers are shown in Table S1.

### 2.14 Proteomic analysis

Livers were lysed with RIPA buffer and 100 μg of protein was digested using the FASP method on 30 kDa spin filters (Millipore) [43]. The eluted peptides were acidified and desalted using in-house made C18 pipette tips (10 μg capacity). Analysis was performed on an Easy nLC-1200 coupled to a ThermoQExactive HF mass spectrometer (Thermo Fisher Scientific) operating in a top 20 mode. The mobile phase was composed of Buffer A (0.1% formic acid) and Buffer B (0.1% formic acid in 80% acetonitrile). Peptides were separated using a PepMap RSLC C18 2 μm, 75 μm × 50 cm column and a PepMap 100 C18 3 μm, 75 μm × 2 cm precolumn with a 2 h gradient of 5%–40% Buffer B. Data were analyzed using MaxQuant (v1.6.10.43) [44] and Perseus [45]. The mass spectrometry proteomics data have been deposited to the ProteomeXchange Consortium via the PRIDE [46] partner repository with the dataset identifier PXD047662.

### 2.15 Western blot analysis

Total protein was extracted from small piece of liver with RIPA buffer (150 mM NaCl, 1% NP-40, 0.5% sodium deoxycholate, 0.1% SDS, and 50 mM Tris, pH 7.8) supplemented with Roche cOmplete™ protease inhibitor cocktail (Sigma–Aldrich). After centrifuging the homogenates, supernatant was collected, and protein concentration was determined by using BCA assay (ThermoFisher Scientific). 40 μg protein was separated by a 4–20% gradient SDS gel, transferred to a PVDF membrane (Millipore), and membrane was blocked for 1 h in 5% milk in TBS-T (50 mM Tris–HCl pH 7.4, 150 mM NaCl, and 0.1% Tween-20) and incubated overnight at 4 °C with primary antibodies: anti-PDK4 (1:500; provided by Dr. Robert A. Harris from the Indiana University School of Medicine (Indianapolis, IN)), anti-α/β Tubulin (1:1000; Cell Signaling, 2148), and secondary HRP-conjugated anti-rabbit IgG (1:2000; Cell Signaling, 7074). Samples were visualized with ECL-Prime and X-ray film (GE health care; Piscataway, NJ) and blots were quantified using ImageJ software (NIH, Fiji).

### 2.16 Histological analyses

A small piece of liver, epididymal WAT (eWAT), inguinal WAT (iWAT), and BAT were isolated and immediately fixed in 10% neutral buffered formalin for 24–48 h at room temperature. After fixation of tissues, samples were processed by the UHN Pathology Research Program Laboratory (Toronto, ON) and embedded in paraffin wax, sectioned, and stained with hematoxylin and eosin (H&E). Images were taken using an EVOS XL Core cell imaging system (Life Technologies, Carlsbad, CA). Adipocyte sizes from iWAT and eWAT were analyzed using a ImageJ software (NIH-Fiji) with Adiposoft plugin for seven or eight images per group [47].

### 2.17 Liver lipid analyses

Liver samples were cut (∼100 mg per piece), frozen in liquid nitrogen, and then stored at −80 °C until extraction. After liver samples were weighed, lipids were extracted from liver in chloroform/methanol (2:1, v/v) using the Folch method [40]. The resulting homogenates were washed once in 50 mM NaCl and centrifuged at 1500×*g* for 30 min, then the organic phase was transferred into new tubes. The organic phase was washed twice in 0.36 M CaCl_2_/methanol and centrifuged at 1500×*g* for 10 min. Afterwards, organic phase was transferred into 5 mL volumetric flasks and brought up to 5 mL with chloroform. Dried aliquots of standards and samples were redissolved in 10 μL of 1:1 chloroform/Triton X-100 and evaporated overnight. Samples were assayed for triglycerides (Infinity, ThermoFisher Scientific) and cholesterol (ThermoFisher Scientific) using colorimetric reagents.

### 2.18 Lipolysis assays

For *in vitro* lipolysis: Differentiated T37i cells were incubated with 25 μM LDT409 for 16 h and then 50 μL of media was collected from the cell culture and cells were lysed for collection of RNA or protein. Free glycerol was measured in the media using the Free Glycerol Reagent (Sigma–Aldrich) and then glycerol levels were normalized to RNA amount.

For *ex vivo* lipolysis: WT (C57Bl/6, 8–9 months old) mice were fed a chow diet or HFD and then intraperitoneally treated with vehicle (5% DMSO and 5% Tween-80 in saline) or 40 mg/kg LDT409 for 14 days. On day 14 of treatment, eWAT, iWAT, and BAT tissues were isolated, cut into ∼70 mg pieces, minced, and incubated with Krebs–Ringer buffer supplemented with bovine serum albumin and 1X penicillin/streptomycin for 4 h at 37 °C, 5% CO_2_ for basal *ex vivo* lipolysis. For β-adrenergic stimulation of lipolysis, tissue pieces were incubated in the presence of 1 μM isoproterenol (Iso; Sigma–Aldrich). Following a 4 h incubation, media was collected, and free glycerol released from the fat depots was measured using a colorimetric Free Glycerol Reagent (Sigma–Aldrich). Glycerol values were normalized by fat explant weights.

### 2.19 Statistical analyses

Results are expressed as means ± SEM. EC_50_ values were calculated using nonlinear regression analysis with GraphPad Prism 8.0. Where appropriate, significance was calculated by one- or two-way ANOVA using Holm-Sidak for multiple comparisons. Data were analyzed using GraphPad Prism 8.0 Software (Graph-Pad, San Diego, CA). *P* < 0.05 was considered statistically significant. For indirect calorimetry studies, statistical analysis and plotting were performed with *CalR* using analysis of covariance (ANCOVA) where appropriate, and the generalized linear model (GLM) where ANCOVA is not appropriate [48].

## 3 Results

### 3.1 LDT409 promotes weight loss in HFD-induced obesity

We first assessed the potency and selectivity of LDT409 for mouse PPARs. We performed a full dose–response curve comparing LDT409 against positive controls for each mouse PPAR (WY, PPARα agonist; Rosi, PPARγ agonist; GW0742, PPARδ agonist) in HEK293 cells using transient transfection assays (Supplemental Fig. 1). LDT409 exhibited balanced partial agonist activity against PPARα and PPARγ with a half-maximal effective concentration (EC_50_) of 0.1 μM (Supplemental Figs. 1B and 1C). LDT409 was also a partial agonist of mouse PPARδ with an EC_50_ of 1 μM (Supplemental Fig. 1D). Taken together, LDT409 was a pan-PPAR agonist with partial agonist activity for mouse PPAR isoforms.

**Figure 1:**
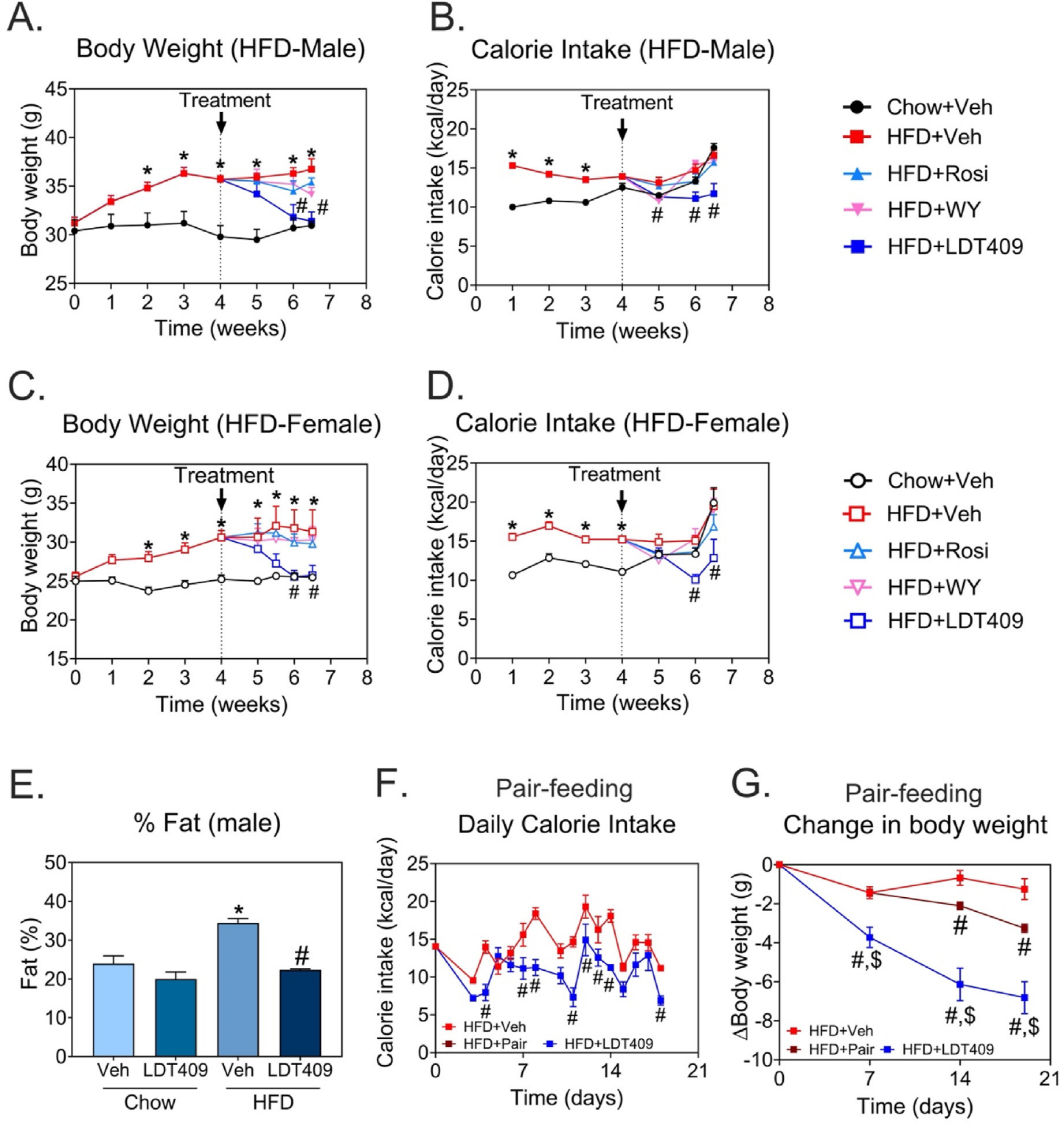
**LDT409 treatment reduces body weight, fat mass and caloric intake in HFD fed mice.** (A–H) Mice with DIO received vehicle, rosiglitazone (Rosi), WY14643 (WY) or LDT409 for 2.5 weeks (18 days). (A) Body weight measurement in male chow-fed and HFD-fed mice for 6.5 weeks, n = 6–8 per group. (B) Daily caloric intake of male mice during the duration of study, n = 6–8 per group. (C) Body weight measurement in female chow-fed and HFD-fed mice for 6.5 weeks, n = 4–5 per group. (D) Daily caloric intake of female mice during the duration of study, n = 4–5 per group. (E) Percentage of fat mass measured by DEXA scan, n = 3–4 per group. (F–G) (F) Daily food intake in HFD-fed mice that received vehicle or 40 mg/kg LDT409 during pair-feeding study, n = 7–8 per group. (G) Body weight changes during the treatments under *ad libitum* or pair-feeding states, n = 7–8 per group. Caloric intake (kcal/day) = Food intake (g diet/day) x caloric value of diet (kcal/g). Data represent the average ± SEM. ^∗^*P* < 0.05 vs Chow + Veh, ^#^*P* < 0.05 vs HFD + Veh, ^§^*P* < 0.05 vs HFD + Pair, using two-way ANOVA with Holm-Sidak correction.

To assess the potential therapeutic effect of LDT409 *in vivo*, 6-month-old C57Bl/6 mice were fed *ad libitum* either a standard chow diet (3 kcal/g of food) or a HFD (4.5 kcal/g of food) for 4 weeks to obtain a mouse model of DIO. As expected, male and female mice fed a HFD increased their body weight ∼25% over chow-fed mice (Figure 1A,C). Mice were treated daily with intraperitoneal injections of vehicle, 10 mg/kg Rosi, 40 mg/kg WY or 40 mg/kg LDT409 for 18 days while continuing the HFD. LDT409 treatment induced significant weight loss over the treatment period (−14% in male and −16% in female mice, *P* < 0.05) such that body weight was normalized back to chow-fed controls after 2.5 weeks. In contrast, Rosi- and WY-treatment in males only decreased body weight by −2.6% and −6%, respectively, both of which were not significantly different from the HFD vehicle-control (Figure 1A). In HFD-fed female mice, Rosi and WY reduced body weight by −2.7% and −1%, respectively (Figure 1C). Notably, LDT409 had no significant impact on body weight in chow-fed male and female mice (Supplemental Figs. 2A and 2D), indicating that LDT409 induces weight loss only in obese mice.

We next measured whether food intake was affected. As expected, due to the higher caloric value of the HFD, HFD-fed mice showed lower food consumption than chow-fed mice (Supplemental Figs. 3A and 3B). When comparing HFD intake in mice treated with vehicle against Rosi, WY or LDT409; only LDT409 treatment decreased food intake (Supplemental Figs. 3A and 3B). This decreased food intake translated into a significantly reduced caloric intake compared to chow- and HFD-fed mice (Figure 1B,D). In contrast, in chow-fed mice, no significant difference in food intake (or caloric intake) was observed with LDT409 treatment (Supplemental Figs. 2B–C and 2E-F). We measured the total body fat composition of chow-fed and HFD-fed male mice that received vehicle or LDT409 treatment by using DEXA. DEXA analysis showed that HFD-fed mice exhibited increased fat content compared to chow-fed control mice (Figure 1E). Consistent with weight loss in LDT409-treated obese mice, there was a significant decrease in body fat content in mice fed HFD, but not chow (Figure 1E). Thus, LDT409 promotes weight loss and decreased food intake in obese mice of both sexes.

### 3.2 LDT409-induced weight-loss is partially explained by decreased food intake

To determine whether the weight-loss effect of LDT409 could be entirely explained by the decrease in food intake seen in LDT409-treated obese mice, we performed a pair-feeding study. *Ad libitum* food intake was measured daily in the LDT409-treated mice, and this amount of food was provided to a ‘pair-fed’ vehicle-treated HFD-fed group alongside the usual *ad libitum* vehicle-treated HFD group (Figure 1F). HFD-fed LDT409- and pair-fed vehicle treated mice thus had a similar food intake which were lower than that of the *ad libitum* HFD-vehicle group (Figure 1F). This caloric restriction, when applied to the pair-fed control mice, resulted in a 5.1% reduction (*P* < 0.05) in overall body weight compared to the HFD-fed control mice fed *ad libitum* (Figure 1G). Notably, with LDT409 treatment, the average weight loss compared to *ad libitum* fed HFD-vehicle controls was 14.5% (*P* < 0.05) (Figure 1G). Collectively, these data indicate that LDT409 promotes weight loss in part through a decrease in food intake, but this mechanism only accounts for 35% of the total weight loss observed.

### 3.3 LDT409 protects from hyperlipidemia and insulin resistance in DIO male mice

**Figure 2:**
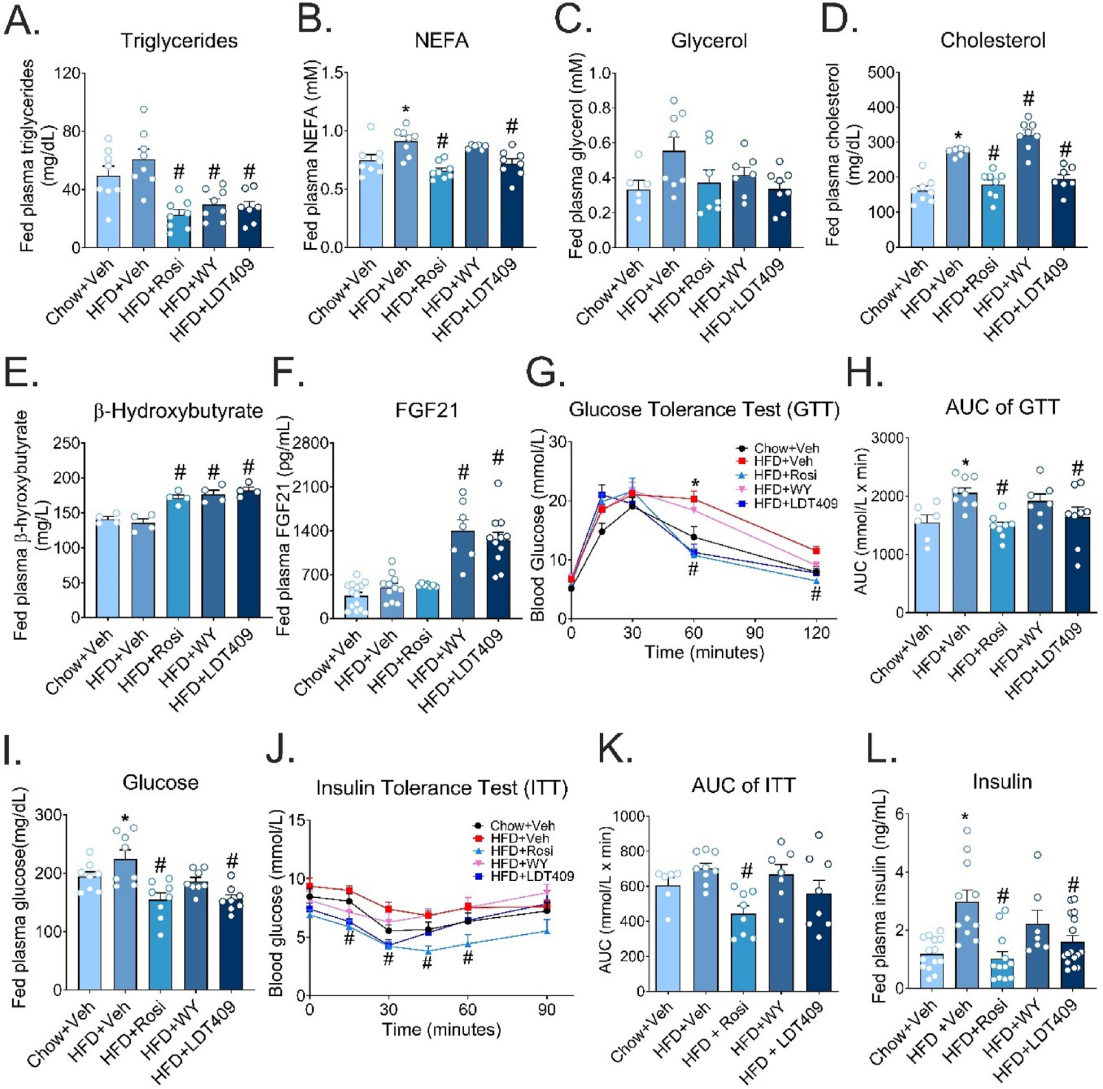
**LDT409 protects against hyperlipidemia and glucose and insulin intolerance in HFD-fed male mice.** (A–H) Measurements of fed plasma triglycerides (A, n = 6–8), NEFA (non-esterified fatty acid) (B, n = 6–8), glycerol (C, n = 7–8), cholesterol (D, n = 6–8), β-hydroxybutyrate (E, n = 4), and FGF21 (Fibroblast growth factor 21) (F, n = 7–14) were obtained from trunk blood at the end of the experiment. (G) Glucose tolerance test (GTT) was performed on day 12 of treatment. After an overnight 16 h-fast, mice were injected with 1 g d-glucose/kg body weight, and tail vein glucose was assayed at 15, 30, 45, 60, and 120 min after injection (n = 6–9). (H) Calculation of area under curve (AUC) from GTT (n = 6–9). (I) Measurement of fed plasma glucose (n = 7–8). (J) Insulin tolerance test (ITT) was performed on day 16 of treatment. After a 4 h-fast, mice were injected with 1 IU/kg body weight, and tail vein glucose was assayed at 15, 30, 45, 60, and 90 min after injection (n = 6–9). (K) Calculation of area under curve (AUC) from ITT (n = 6–9). (L) Measurement of fed plasma insulin (n = 7–16). Data represent the average ± SEM. ^∗^*P* < 0.05 vs Chow + Veh, ^#^*P* < 0.05 vs HFD + Veh, using one-way (panels A–F, H, I, K, L) or two-way ANOVA (panel G, J) with Holm-Sidak correction.

Next, we assessed whether weight loss induced by LDT409 would be accompanied by an improvement in lipid and glucose metabolism. Plasma triglyceride and non-esterified fatty acid (NEFA) levels were significantly lower in LDT409-treated mice than in the HFD control mice (Figure 2A,B). No significant changes in plasma glycerol levels were observed between the groups (Figure 2C). Surprisingly, LDT409 and Rosi treatment markedly reduced plasma cholesterol levels compared to vehicle, even though all were fed the same cholesterol-rich HFD (0.2% cholesterol w/w) (Figure 2D). In contrast, cholesterol levels were elevated in WY-treated HFD-fed mice (Figure 2D), possibly due to increased secretion of ApoB-100 containing low density lipoproteins (LDL/IDL), as has been reported previously by WY [49].

To determine whether LDT409 was activating PPARα target genes in the liver, plasma β-hydroxybutyrate and fibroblast-growth factor 21 (FGF21) levels were measured. LDT409, WY, and Rosi each significantly increased plasma β-hydroxybutyrate levels as a marker of ketogenesis compared to chow and HFD vehicle control groups (Figure 2E). FGF21 is a direct target of PPARα and is involved in the regulation of energy homeostasis [[50], [51], [52], [53]]. As expected, circulating FGF21 levels were increased 2.5-fold with LDT409 and 2.8-fold with WY treatment in HFD-fed mice compared to HFD-fed vehicle controls (Figure 2F).

To assess the influence of LDT409 on glucose homeostasis, we performed an intraperitoneal glucose and insulin tolerance test (GTT and ITT) in male mice on days 12 and 16 of treatment, respectively. LDT409 treated mice showed a significant improvement in glucose disposal at 60 and 120 min (Figure 2G) and this was reflected in the lower area under the curve (AUC) of the GTT compared to vehicle treatment (Figure 2H). The benefit of LDT409 on glucose homeostasis was maintained in the random-fed state, reflected by the lower fed plasma glucose levels compared to HFD-vehicle (Figure 2I). LDT409-treated mice exhibited improved insulin tolerance with blood glucose decreasing by 42% within 30 min compared to the 20% decrease by HFD-fed control mice (Figure 2J). Meanwhile, LDT409 treatment exhibited a non-significant (−21%) decrease in AUC of ITT (Figure 2K). In contrast, as expected, Rosi showed a strong insulin-sensitizing effect with a significantly decreased AUC of ITT compared to HFD-fed mice (Figure 2J,K). Similarly, circulating insulin concentrations were lower in LDT409- and Rosi-treated obese mice than vehicle-treated HFD-fed mice (Figure 2L). Taken together, our results indicate LDT409 exhibits both lipid and glucose lowering effects in obese mice. Therefore, these results provide further evidence that LDT409 plays an important role in protecting mice from the effects of diet-induced hyperlipidemia and improves glucose homeostasis. One caveat related to the interpretation of these data is that the mice were dosed based on total body weight as opposed to lean body weight which could influence the magnitude of the effects observed.

### 3.4 LDT409 ameliorates hepatic steatosis in HFD-fed mice

**Figure 3:**
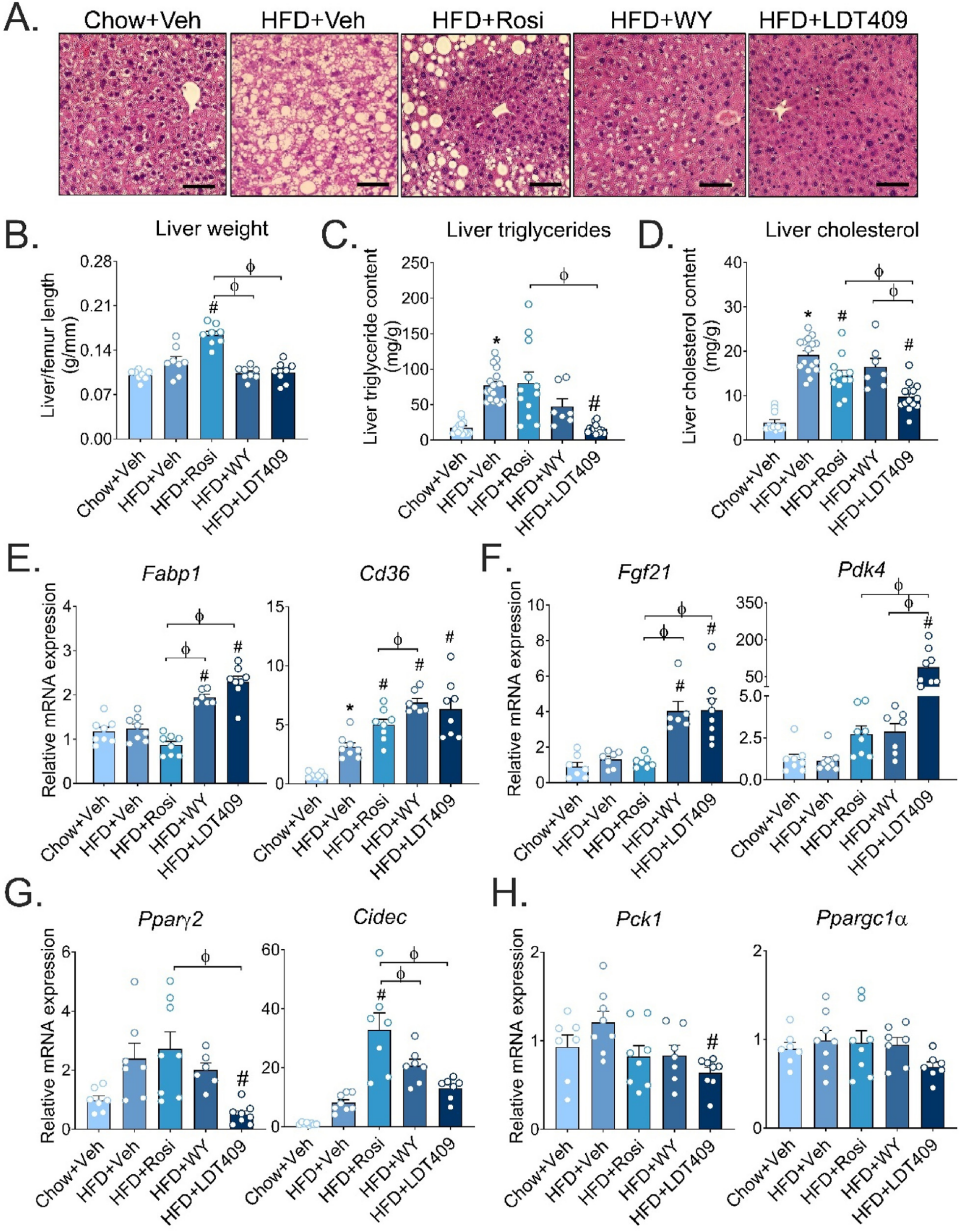
**LDT409 ameliorates hepatic steatosis in HFD-fed male mice.** (A–H) Mice with DIO received vehicle, rosiglitazone (Rosi), WY14643 (WY) or LDT409 for 2.5 weeks (18 days). (A) Representative liver sections with H&E staining, n = 3–4 per group. Scale bar, 200 μm. (B) Liver weight at time of harvest, n = 8 per group. (C and D) Measurement of liver triglycerides (C) and liver cholesterol (D) from fed mice, n = 7–16 per group. (E–H) Quantitative real-time PCR analysis for expression of fatty acid uptake genes (*Fabp1* and *Cd36*) (E), fatty acid oxidation genes (*Fgf21* and *Pdk4*) (F), fat droplet formation-associated genes (*Pparγ2* and *Cidec*) (G), and gluconeogenic genes (*Pck1* and *Ppargc1α*) (H), n = 7–8 per group. Data represent the average ± SEM. ^∗^*P* < 0.05 vs Chow + Veh, ^#^*P* < 0.05 vs HFD + Veh, ^φ^*P* < 0.05 vs indicated group using one-way ANOVA with Holm-Sidak correction.

Histochemical analyses were carried out on liver tissues of mice to morphologically examine the effects of LDT409 on hepatic lipid accumulation. Significant lipid droplets were observed in livers of HFD-fed mice treated with vehicle or Rosi compared to chow (Figure 3A). WY treatment showed reduced lipid droplets compared to HFD-vehicle, whereas virtually no lipid droplets were detected by histology with LDT409-treatment (Figure 3A). HFD feeding increased liver weight by ∼20% compared to chow-fed mice (Figure 3B). Notably, Rosi significantly increased liver weight by 63% and 35% compared to chow-fed and HFD-fed mice, respectively, whereas WY and LDT409 treatment had no impact on liver weight compared to HFD (Figure 3B). This effect of Rosi on liver weight has been previously attributed to liver PPARγ agonism promoting hepatic lipid droplet formation [54,55]. Biochemical analysis of liver lipid extracts found that triglyceride and cholesterol levels were significantly decreased with LDT409 (by 4.8-fold and 2-fold, respectively) compared to HFD-fed control (Figure 3C,D), in line with the representative images of liver H&E (Figure 3A). Interestingly, LDT409 significantly upregulated genes related to fatty acid uptake: fatty acid-binding protein 1 (*Fabp1*) and fatty acid translocase (*Cd36*), consistent with PPARα activation (Figure 3E). From liver proteomic analysis, LDT409 significantly increased PPARα target proteins including CYP4A14, ACOX1, ACADM, ECl1, and ECl2 that are involved in the oxidation of fatty acids (Supplemental Figs. 4A–E). The mRNA expression of direct PPARα-target genes such as *Fgf21* and pyruvate dehydrogenase kinase 4 (*Pdk4*), was also increased by LDT409 treatment in HFD-fed mice (Figure 3F). Indeed, upregulation of *Fgf21* was strongly correlated with the circulating plasma FGF21 levels (Figures 2F and 3F). Expression of *Pdk4* was increased as expected by WY and Rosi (∼2.5-fold each); however, *Pdk4* showed a dramatic 78-fold induction with LDT409 (*P* < 0.05) compared to HFD-vehicle (Figure 3F). PDK4 is known to inhibit the pyruvate dehydrogenase complex and promote fatty acid utilization but this is tightly regulated with a protein half-life of 1 h [56]. By Western blot we found that PDK4 protein levels were increased 2.5-fold in LDT409 treatment compared to chow and HFD-fed vehicle-treated mice (Supplemental Fig. 5). Cell death-inducing DFFA-like effector c (*Cidec*, also known as *Fsp27*) is a gene important for fat droplet formation and is upregulated through activation of hepatocyte PPARγ2 [57]. As expected, Rosi strongly upregulated *Cidec,* while this gene was not significantly increased by LDT409 compared to HFD-vehicle (Figure 3G). Lastly, the only significant difference in the expression of gluconeogenic-targeted genes, including phosphoenolpyruvate carboxykinase (*Pck*) and peroxisome proliferator-activated receptor-gamma coactivator alpha 1 (*Ppargc1α*), was a decrease in *Pck1* by LDT409 when compared to HFD-vehicle (Figure 3H). These results suggest that with HFD-feeding LDT409, despite being a substrate of both PPARα and PPARγ, plays a dominant role in promoting fatty acid oxidation, resulting in a reduction in hepatic steatosis.

### 3.5 LDT409 activates “browning” in inguinal white adipose tissue

**Figure 4:**
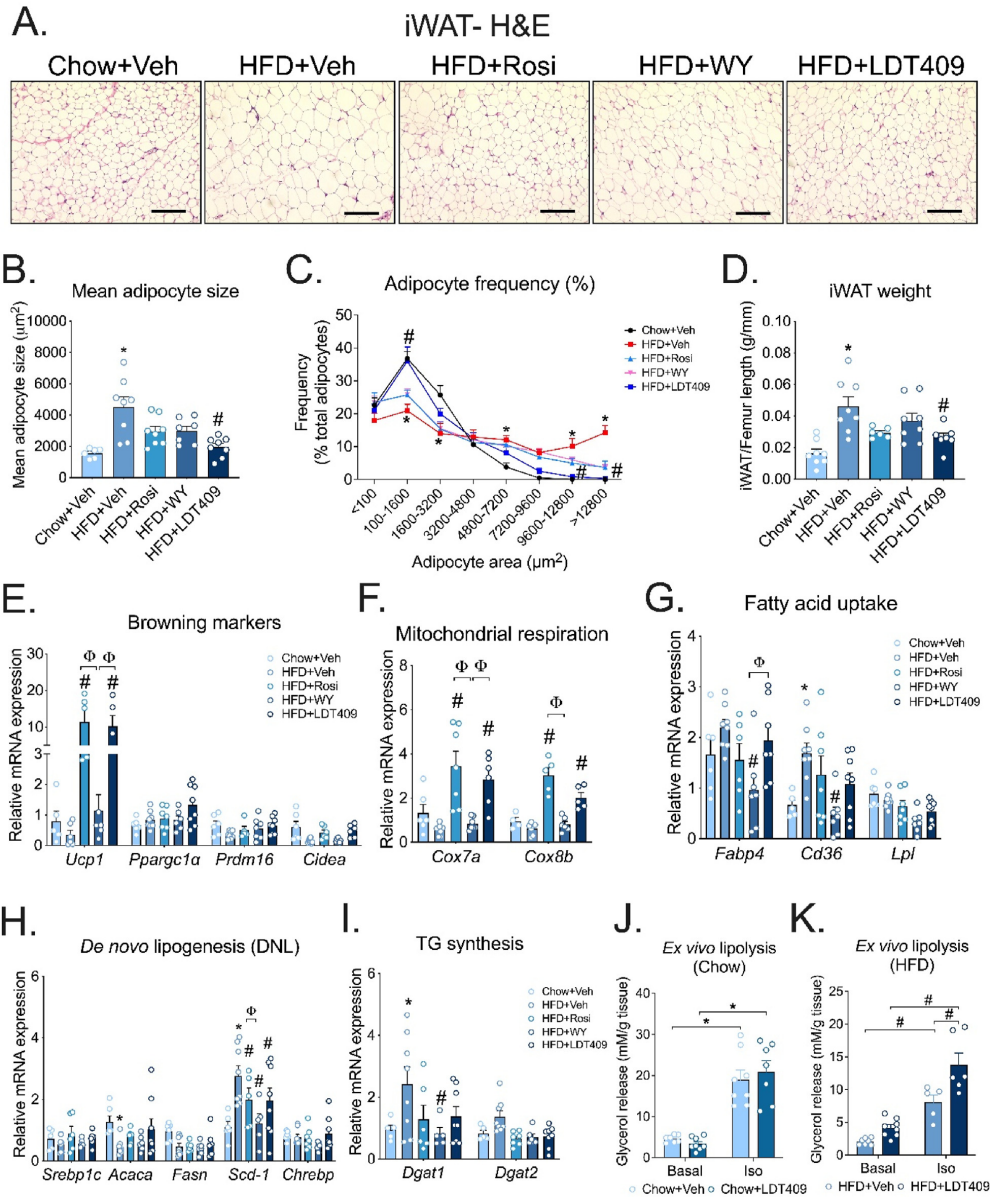
**LDT409 activates browning of inguinal white adipose tissue (iWAT).** (A–I) Mice with DIO received vehicle, rosiglitazone (Rosi), WY14643 (WY) or LDT409 for 18 days. (A) Representative image of inguinal adipose tissue (iWAT) sections with H&E staining, n = 6–8 per group. Scale bar, 200 μm. (B) Mean adipocyte cell size, n = 6–8 per group. (C) Frequency distribution of different-sized adipocytes of iWAT, n = 6–8 per group. (D) iWAT weight at time of harvest, n = 6–8 group. (E–I) Quantitative real-time PCR analysis for expression of (E) browning marker genes (*Ucp1, Ppargc1α, Prdm16,* and *Cidea*), (F) mitochondrial respiration genes (*Cox7a* and *Cox8b*), (G) fatty acid uptake genes (*Fabp4, Cd36,* and *Lpl*), (H) *de novo* lipogenesis (DNL) genes (*Srebp1c*, *Acaca*, *Fasn*, *Scd-1*, and *Chrebp*) and (I) TG synthesis genes (*Dgat1* and *Dgat2*) n = 5–8 per group. (J–K) *Ex vivo* lipolysis in iWAT explants from chow-fed and HFD-fed mice treated with vehicle or 40 mg/kg LDT409. Lipolysis was induced with 1 μM Iso, n = 5–8. These data represent the average ± SEM. ^∗^*P* < 0.05 vs Chow + Veh, ^#^*P* < 0.05 vs HFD + Veh ^φ^*P* < 0.05 vs indicated group using one-way ANOVA (panels B, D-I) or two-way ANOVA (panels C, J, K) with Holm-Sidak correction.

PPARγ activation (through Rosi) is well-known to promote browning of WAT and contributes to its anti-diabetic actions [58]. In this study, we compared the browning effects of Rosi with that of our partial PPARγ agonist, LDT409 [37]. Consistent with the weight gain observed, HFD-feeding induced an enlargement in the overall size of adipocytes within iWAT (Figure 4A). In contrast, we observed smaller adipocytes and measured a reduction in the average size of adipocytes in iWAT of the LDT409-treated vs. vehicle-treated mice (Figure 4A,B). Meanwhile, an increase in the frequency of smaller adipocytes was observed in LDT409-treated compared to control mice (Figure 4C). LDT409 also decreased iWAT weight in HFD-fed mice (Figure 4D). Gene expression analysis showed that LDT409 and Rosi significantly increased the expression of the thermogenic gene *Ucp1* compared to HFD-fed control (Figure 4E). Additionally, the mRNA expression of mitochondrial oxidative genes, *Cox7a* and *Cox8b*, was robustly increased by LDT409 and Rosi in HFD-fed mice compared to chow-fed and HFD-fed control mice (Figure 4F).

We then measured expression of fatty acid uptake genes (*Fapb4, Cd36,* and *Lpl*). The mRNA expression of *Cd36* was only highly upregulated in HFD-fed mice with vehicle treatment (Figure 4G); in contrast, WY treatment led to downregulation of *Cd36* expression compared to vehicle-treated or Rosi-treated HFD-fed mice (Figure 4G). We found that LDT409 had no significant impact on mRNA expression of genes associated with fatty acid uptake (Figure 4G) or *de novo* lipogenesis (*i.e., Srebp1c, Acaca, Fasn,* and *Chrebp*) (Figure 4H). Interestingly, HFD-upregulated *Scd-1* gene was significantly downregulated by LDT409 treatment (Figure 4H). In adipocytes, metabolic energy is stored as triacylglycerols and the acyl-CoA: diacylglycerol acyltransferase (DGAT) enzymes, DGAT1 and DGAT2, are involved in triacylglycerol synthesis (TG) [59]. Gain and loss of function studies have shown a critical role for DGAT1 in adipocyte TG storage [[60], [61], [62], [63]]. We found that HFD feeding upregulated the expression of *Dgat1* and this was prevented by LDT409 (Figure 4I).

We tested the impact of LDT409 on iWAT lipolysis and found no change in glycerol release at basal levels between the chow-fed and HFD-fed groups (Figure 4J,K). Notably, there was no impact of LDT409 on lipolysis in chow-fed fat explants following lipolytic stimulation by the β-1/2 adrenergic agonist Iso (Figure 4J). *Ex vivo* lipolysis also showed that LDT409 in HFD-fed mice was sufficient to augment the presence of free glycerol in adipocyte supernatant after Iso treatment (Figure 4K). These data suggest that LDT409 promotes browning of adipose tissues and enhances the sensitivity of iWAT to lipolytic stimulation in HFD-fed mice, which could contribute to weight loss.

### 3.6 LDT409 promotes the formation of small adipocytes and protects against HFD-induced obesity and inflammation in epidydimal white adipose tissue

**Figure 5:**
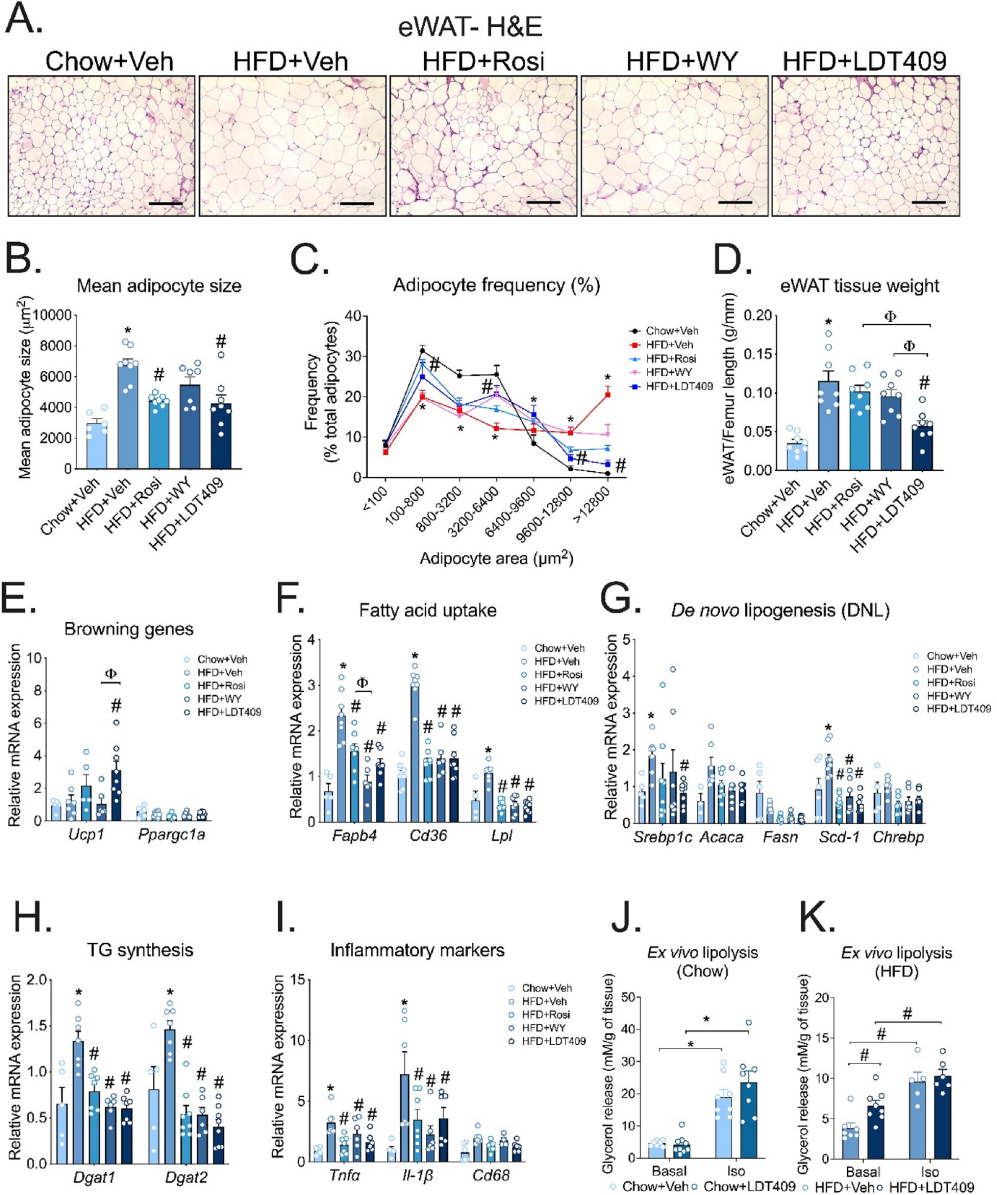
**LDT409 treatment generates smaller adipocytes and protects against weight gain in epidydimal depot.** (A–I) Mice with DIO received vehicle, rosiglitazone (Rosi), WY14643 (WY) or LDT409 for 18 days. (A) Representative image of epididymal adipose tissue (eWAT) sections with H&E staining, n = 6–8 per group. Scale bar, 200 μm. (B) Mean adipocyte cell size, n = 6–8 per group. (C) Frequency distribution of different-sized adipocytes of eWAT, n = 6–8 per group. (D) eWAT weight at time of harvest, n = 6–8 group. (E–I) Quantitative real-time PCR analysis for expression of (E) browning marker genes (*Ucp1, Pgc1α*), (F) fatty acid uptake genes (*Fabp4, Cd36* and *Lpl*), (G) *de novo* lipogenesis (*Srebp1c*, *Acaca, Fasn*, *Scd-1*, and *Chrebp*), (H) TG synthesis (*Dgat1* and *Dgat2*), and (I) pro-inflammatory marker genes (*Tnfα*, *Il-1β*, and *Cd68*), n = 6–8 per group. (J–K) *Ex vivo* lipolysis in eWAT explants from chow-fed and HFD-fed mice treated with vehicle or 40 mg/kg LDT409. Lipolysis was stimulated with 1 μM Iso, n = 5–8. These data represent the average ± SEM. ^∗^*P* < 0.05 vs Chow + Veh, ^#^*P* < 0.05 vs HFD + Veh, ^φ^*P*<0.05 vs indicated group using one-way ANOVA (panels B, D-I) or two-way ANOVA (panels C, J, K) with Holm-Sidak correction.

Histologic analysis of eWAT by H&E staining (Figure 5A) revealed that LDT409 and Rosi treatment resulted in the formation of demonstrably smaller adipocytes compared to HFD-fed control mice. This was confirmed by the quantification of mean adipocyte size (Figure 5B) and by the distribution of adipocyte area (Figure 5C). While Rosi and LDT409 both generated smaller adipocytes of the same mean size, LDT409 was also able to decrease eWAT tissue weight at the end of the study (Figure 5D). These data suggest that LDT409 causes a shrinkage in eWAT fat mass primarily due to the reduced size of the adipocytes vs. Rosi which reduced adipocyte size but likely also promoted adipocyte differentiation [64]. WY had no significant effect on adipocyte mean size or distribution (Figure 5A–D). Of interest, the beige/brown adipocyte marker *Ucp1* was upregulated in response to Rosi and LDT409 treatment (Figure 5E), while *Ppargc1α* gene was not changed under any condition (Figure 5E). The mRNA expression of fatty acid uptake genes (*Fabp4*, *Cd36* and *Lpl*) was significantly downregulated with LDT409, Rosi and WY compared to HFD-vehicle control (Figure 5F). Additionally, LDT409 treatment significantly suppressed mRNA expression of genes involved in *de novo* lipogenesis (*i.e., Srebp1c*, *Scd-1*) (Figure 5G). We found that HFD feeding increased the expression of *Dgat1* and *Dgat2*; and LDT409, Rosi and WY significantly reduced expression of these genes (Figure 5H).

Furthermore, as expected, HFD triggered a significant upregulation of pro-inflammatory genes, including *Tnfα*, and *Il-1β* compared to chow-fed mice (Figure 5I). Treatment with Rosi, WY or LDT409 significantly suppressed adipose tissue inflammatory gene expression (Figure 5I). Next, we assessed whether LDT409 could impact inflammatory cell gene expression from macrophages directly. Consistent with the anti-inflammatory effect observed in adipose tissue, LDT409 strongly downregulated LPS-induced inflammatory gene responses (*Tnfα*, *Il-1β*, and *Mcp1*) in RAW264.7 macrophage-like cells (Supplemental Fig. 6). Together with decreased adipocyte size, decreased inflammation in adipose tissue will promote insulin sensitivity in adipose.

Given the decrease in insulin levels in LDT409-treated DIO mice, we also hypothesized that LDT409 could affect WAT lipolysis. To explore this notion, we initially analyzed secretion rates of glycerol from eWAT explants in chow and HFD-fed mice with vehicle or LDT409 treatment. Although no difference in basal glycerol release was observed between the chow-fed mice groups (Figure 5J), LDT409 significantly increased basal glycerol release in HFD-fed mice (Figure 5K). When explants were treated with Iso, no differences were observed in glycerol release irrespective of the diet (Figure 5J,K), suggesting that LDT409 is promoting basal lipolysis only under HFD-feeding. These data support the idea that LDT409 protects against weight gain by decreasing fatty acid uptake and storage while at the same time promoting adipocyte lipolysis.

### 3.7 LDT409 treatment enhances thermogenic capacity of brown adipocytes in DIO mice

**Figure 6:**
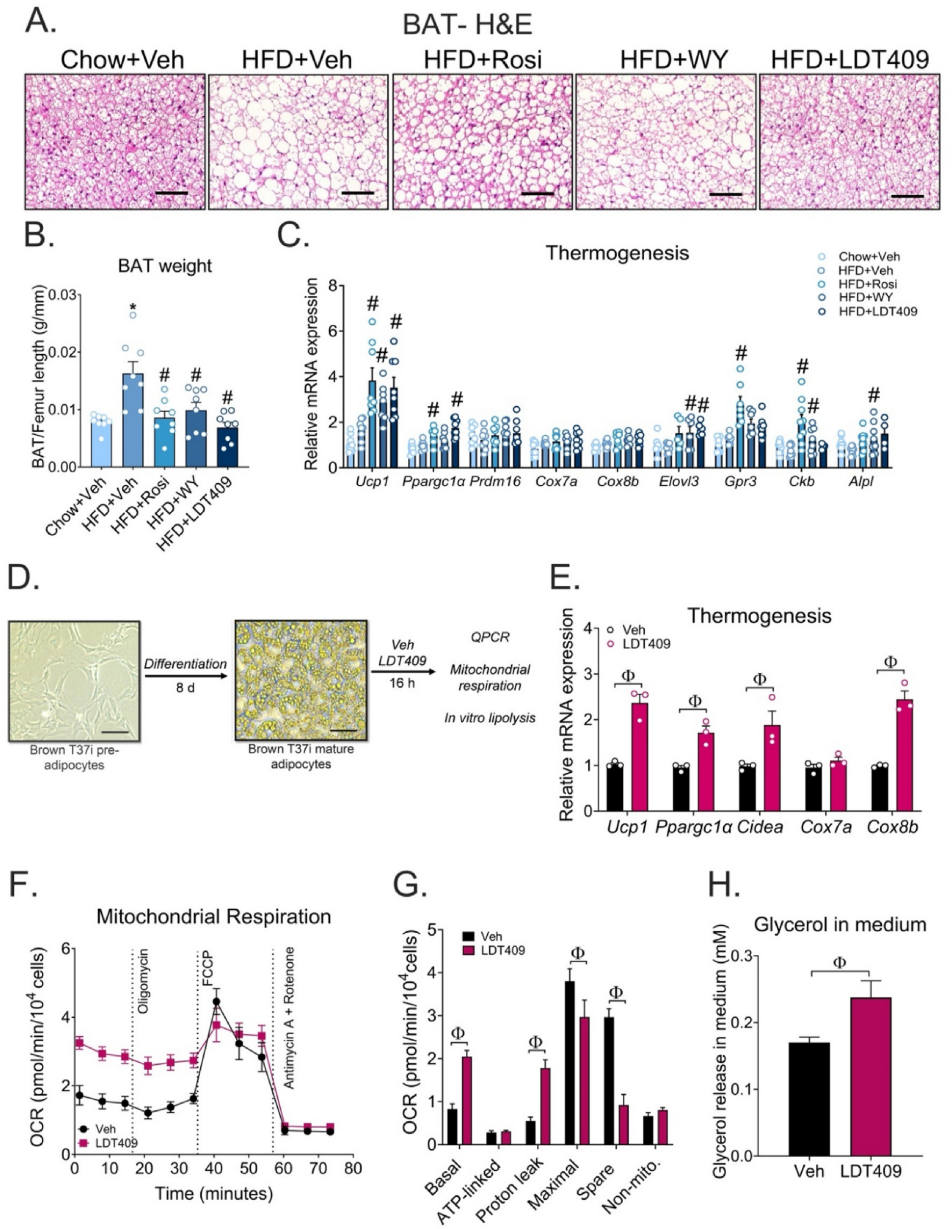
**LDT409 treatment enhances thermogenic capacity of brown adipocytes in DIO mice.** (A–C) Mice with DIO received vehicle, rosiglitazone (Rosi), WY14643 (WY) or LDT409 for 18 days. (A) Representative image of brown adipose tissue (BAT) sections with H&E staining, n = 7–8 per group. Scale bar, 200 μm. (B) BAT weight at time of harvest, n = 6–8 each group. (C) Quantitative real-time PCR analysis for expression of thermogenic genes (*Ucp1*, *Ppargc1α, Prdm16, Cox7a*, *Cox8b*, *Elovl3*, *Gpr3, Ckb, Alpl*), n = 7–8 per group. (D) Representative images of pre-adipocytes (undifferentiated T37i) and T37i brown adipocytes differentiated in the presence of 2 nM triiodothyronine (T_3_) and 20 nM insulin. Scale bar, 200 μm. (E) Quantitative real-time PCR analysis of genes implicated in thermogenesis (*Ucp1*, *Ppargc1α, Cidea, Cox7a,* and *Cox8b*) in differentiated T37i brown adipocytes in absence or presence of 25 μM LDT409 for 16 h, n = 3. (F) Oxygen consumption rates (OCR) following the Mito Stress Test (MST) assay in the differentiated T37i brown adipocytes in the absence or presence of LDT409 for 16 h, n = 7–8. (G) Basal, ATP-linked, proton leak, maximal, spare, and non-mitochondrial respiration rates were calculated from the Seahorse MST assay performed in (F). (H) *In vitro* lipolysis in T37i brown adipocytes, treated with LDT409 for 16 h, n = 3. These data represent the average ± SEM. ^∗^*P* < 0.05 vs Chow + Veh, ^#^*P* < 0.05 vs HFD + Veh, using one-way ANOVA with Holm-Sidak correction or ^φ^*P* < 0.05 vs indicated group using t-test.

Thermogenic activation of BAT is positively correlated with improvement of lipid metabolism [65,66], insulin sensitivity [66,67], and weight loss [68,69]. Histological sections of interscapular BAT revealed enhanced accumulation of lipid droplets in HFD-fed control mice compared to chow-fed control mice (Figure 6A). Intriguingly, these large lipid droplets were decreased in size with Rosi, WY, and LDT409 treatment, but the most dramatic changes relative to the HFD-fed condition were in the LDT409-treated BAT sections (Figure 6A). In agreement with the histology, the weight of the BAT depot was significantly decreased by Rosi, WY, and LDT409 (Figure 6B). We also found a significant increase in the mRNA levels of thermogenic gene *Ucp1* in HFD-fed mice treated with Rosi, WY or LDT409 (Figure 6C). Additionally, we observed that Rosi, WY or LDT409 selectively upregulated other thermogenic genes. For example, Rosi significantly increased *Ppargc1a*, *Gpr3*, and *Ckb* expression; WY upregulated *Elovl3*, *Ckb* and *Alpl* expression; while LDT409 increased *Ppargc1a* and *Elovl3* expression in HFD-fed mice (Figure 6C).

We next investigated whether brown adipocytes could respond cell autonomously to LDT409 by using the T37i cells as an *in vitro* model of mouse brown adipocytes [42]. T37i pre-adipocytes were differentiated into mature brown adipocytes using thyroid hormone, as shown in Figure 6D. Consistent with our *in vivo* data, LDT409 significantly increased the expression of genes involved in thermogenesis (*Ucp1*, *Ppargc1a*, *Cidea*, and *Cox8b*) (Figure 6E) and fatty acid oxidation (*Acadl*, *Cpt1*, and *Pdk4*) (Supplemental Fig. 7). To test whether LDT409-induced changes in mitochondrial respiration, we performed a Mito Stress Test, measuring the oxygen consumption rate in T37i cells after 16 h-LDT409 incubation. Notably, LDT409-treated cells exhibited higher basal oxygen consumption than vehicle-treated control cells (Figure 6F). Specifically, with LDT409 treatment, oxygen consumption associated with basal respiration and proton leak were increased 2.5-fold and 3.2-fold, respectively (*P* < 0.05) (Figure 6G). In contrast, no differences in ATP-linked respiration were observed between the groups (Figure 6G). Additionally, in the presence of 25 μM LDT409 for 16 h, the glycerol released in the medium was significantly increased in T37i brown adipocytes (Figure 6H), supporting the idea that LDT409 induces lipolysis to promote uncoupling. However, we note that acute treatment of T37i cells with LDT409 (using the injection ports of the Seahorse instrument) did not stimulate oxygen consumption. In addition, treatment with LDT409 did not stimulate lipolysis within a 1 h period (data not shown). These findings suggest LDT409 can act cell-autonomously in brown adipocytes in a time-dependent manner to increase lipolytic and thermogenic signaling pathways which could contribute to the weight loss we observed.

### 3.8 Potential mechanisms underlying weight loss in LDT409-treated DIO mice

**Figure 7:**
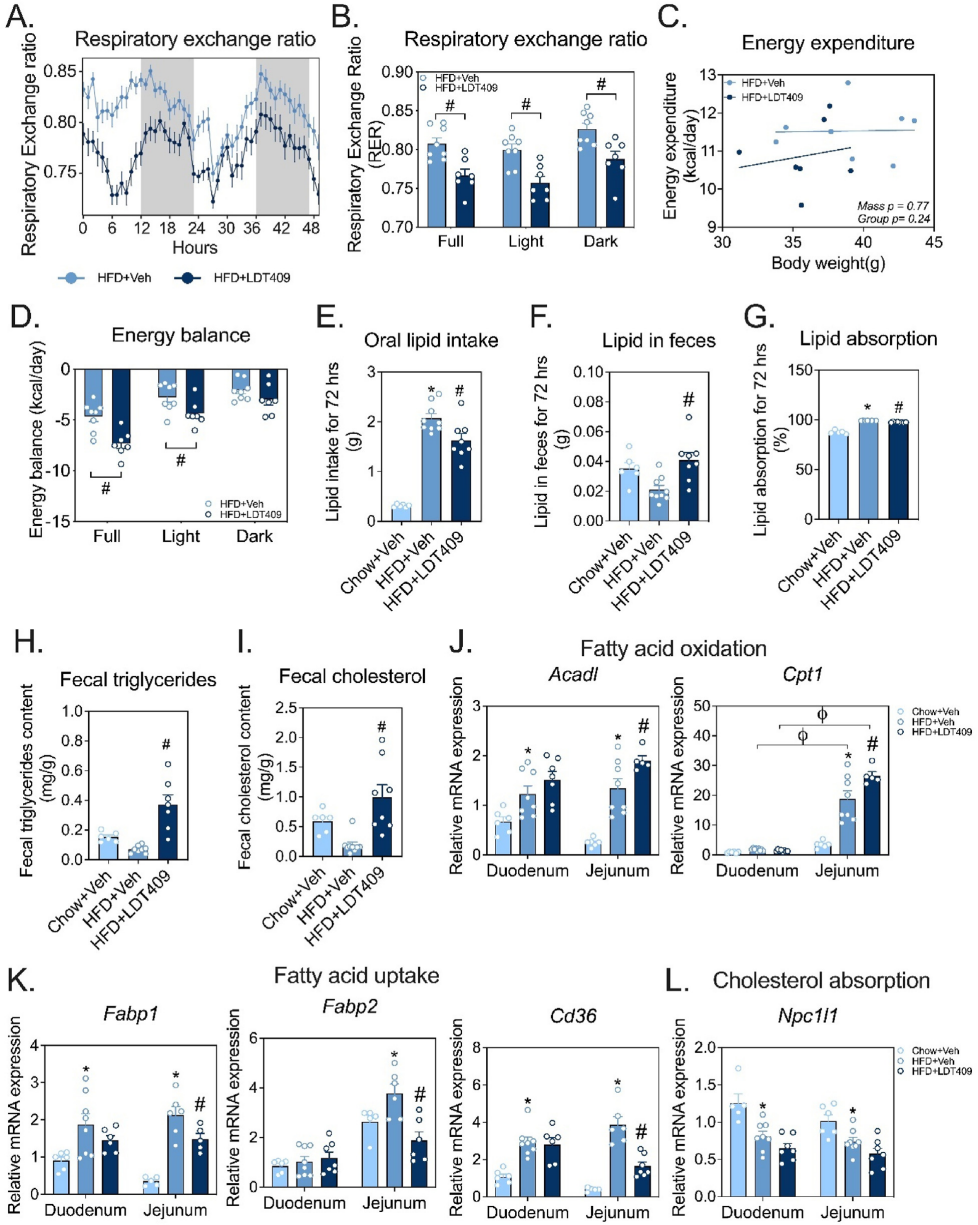
**LDT409 increases fat utilization and decreases intestinal lipid absorption contributing to overall weight loss in HFD-fed mice.** (A–L) Mice with DIO received vehicle or LDT409 treatment for 18 days. (A–B) Respiratory exchange ratio (RER) of HFD-fed mice received vehicle or 40 mg/kg LDT409 for 48 h, n = 7–8. (C) Regression plot of energy expenditure from HFD-fed mice treated with vehicle or 40 mg/kg LDT409 for 48 h, n = 7–8. (D) Energy balance of HFD-fed mice received vehicle or 40 mg/kg LDT409 for 48 h, n = 7–8. (E–I) Oral dietary lipid intake from the mice treated with vehicle or 40 mg/kg LDT409 between day 13 and day 16 of treatment, n = 6–8. (F) Intestinal lipid absorption, n = 6–8. (G) Lipid in feces, n = 6–8. (H–I) Fecal triglycerides and cholesterol content levels, n = 6–8. (J–L) Quantitative real-time PCR analysis from duodenum and jejunum of (J) fatty acid oxidation genes (*Acadl and Cpt1*), (K) fatty acid uptake genes (*Fabp1, Fabp2,* and *Cd36*) and (L) cholesterol absorption gene (*Npc1l1*). These data represent the average ± SEM. ^∗^*P* < 0.05 vs Chow + Veh, ^#^*P* < 0.05 vs HFD + Veh, ^φ^*P*<0.05 vs indicated group using one-way or two-way ANOVA with Holm-Sidak correction.

To assess the impact of LDT409 on substrate utilization and energy expenditure, indirect calorimetry was performed on mice placed in metabolic cages for a period of 72 h. Following a 24 h acclimation period, metabolic parameters, including respiratory exchange ratio (RER), energy expenditure (EE), energy balance, and physical activity, were measured for the remaining 48 h. The RER value provides an estimate of substrate utilization preference, with an RER of 0.7 indicating primarily lipid utilization, while an RER of 1.0 indicates high glucose utilization. Importantly, LDT409-treated DIO mice displayed a lower RER level than vehicle-treated DIO control mice (Figure 7A), during both the light and dark cycles (Figure 7B), implying increased overall fatty acid oxidation in LDT409-treated HFD-fed mice. Interestingly, no differences in RER values were observed between chow-fed mice receiving vehicle or LDT409 treatment (Supplemental Figs. 8A and 8B). Furthermore, we assessed whether LDT409 impacted overall EE using analysis of covariance (ANCOVA) [70], controlling for the effect of body weight on EE. A regression plot of 48 h average EE versus body weight showed no significant correlation to body weight in HFD-fed mice and no changes between vehicle and LDT409 treatment (Figure 7C, Supplemental Figs. 9A–C). In contrast, ANCOVA revealed a positive correlation between EE and body mass in chow-fed mice which was not impacted LDT409 treatment (Supplemental Figs. 8C–E). Likewise, there were no alterations in ambulatory and locomotor activities between LDT409-treated and vehicle-treated HFD-fed mice during light- and dark-cycles (Supplemental Figs. 9E–F and 9H–I). However, when considering the overall energy balance, which is EE subtracted from energy intake, we observed a significant decrease in LDT409-treated mice during the light cycle, which persisted when analyzed over the full cycle (Figure 7D). This contributed to a negative energy balance (Figure 7D), thereby resulting in the weight-loss observed for LDT409-treated obese mice. In chow-fed mice, we observed no significant differences in EE, food intake, energy balance or total physical activity between vehicle or LDT409 treatment (Supplemental Figs. 8F–I). Overall, these results show that LDT409 significantly increased fat utilization and promoted negative energy balance only in HFD-fed obese mice.

In addition to considering energy intake and EE, the efficiency of energy absorption from the gut is another potential contributor to weight loss. We hypothesized that the decrease in plasma lipids and body weight by LDT409 in HFD-fed mice could be attributed to increased flow of dietary lipids in the feces. Each mouse was individually housed and treated daily with either vehicle or LDT409 for 18 days; feces was collected for 72 h between day 7–10 and day 13–16 of treatment. As expected, the extent of oral lipid consumed by the mice on HFD was increased compared to chow due to the high percentage of fat within the diet (42% kcal vs 12% kcal; Figure 7E and Supplemental Fig. 10A). The extent of lipid excretion into the feces was unchanged with HFD (Figure 7F), resulting in 99% efficient lipid absorption in HFD-fed mice compared to 88% in chow-fed mice (Figure 7G). With LDT409 treatment, oral lipid intake was significantly decreased (Figure 7E), in line with our previous finding of decreased food intake; however, fecal lipid output was significantly increased, resulting in a significant decrease in the efficiency of intestinal lipid absorption with LDT409 compared to vehicle (97.5% vs 99.1%, *P* < 0.05; Figure 7F–G). In agreement with the gravimetric analysis of bulk lipids extracted from feces, LDT409-treated HFD-fed mice showed higher levels of triglycerides and cholesterol in the feces (Figure 7H–I, Supplemental Figs. 10G–H). Moreover, HFD significantly increased the PPARα-regulated fatty acid oxidation genes (*Acadl* and *Cpt1*) in the gut (Figure 7J). Intriguingly, we observed that *Cpt1* expression was highly induced in the jejunum compared to the duodenum in response to HFD (Figure 7J). Furthermore, LDT409 significantly upregulated the expression of fatty acid oxidation genes in jejunum indicating that increased oxidation is not only happening in the liver but also the gut. In the duodenum and jejunum, the mRNA expression of fatty acid uptake genes (*Fabp1* and *Cd36*) was significantly upregulated by HFD, likely contributing to increased lipid absorption (Figure 7K). The jejunum is the primary region of small intestine for lipid digestion and absorption [71] and LDT409 treatment suppressed *Fabp1, Fabp2,* and *Cd36* expression compared to HFD control (Figure 7K). We also found that expression of the cholesterol uptake protein Niemann-Pick C1-Like 1 (*Npc1l1*) was significantly downregulated by HFD feeding and tended to decrease further with LDT409 in duodenum and jejunum (Figure 7L). These data suggest that LDT409 plays a role in the regulation of intestinal lipid absorption and utilization. However, despite the statistically significant impact of LDT409 on lipid absorption; when we consider the absolute energetic cost of this loss, its contribution to the overall differences in negative energy balance is minimal (*i.e.,* the difference in fecal lipids is equivalent to ∼0.2 kcal). Thus, LDT409 promotes negative energy balance in HFD-fed mice through a combination of decreased food intake and increased fat utilization, with a minor contribution from increased fecal lipid excretion.

### 3.9 LDT409 increases energy expenditure at thermoneutrality

**Figure 8:**
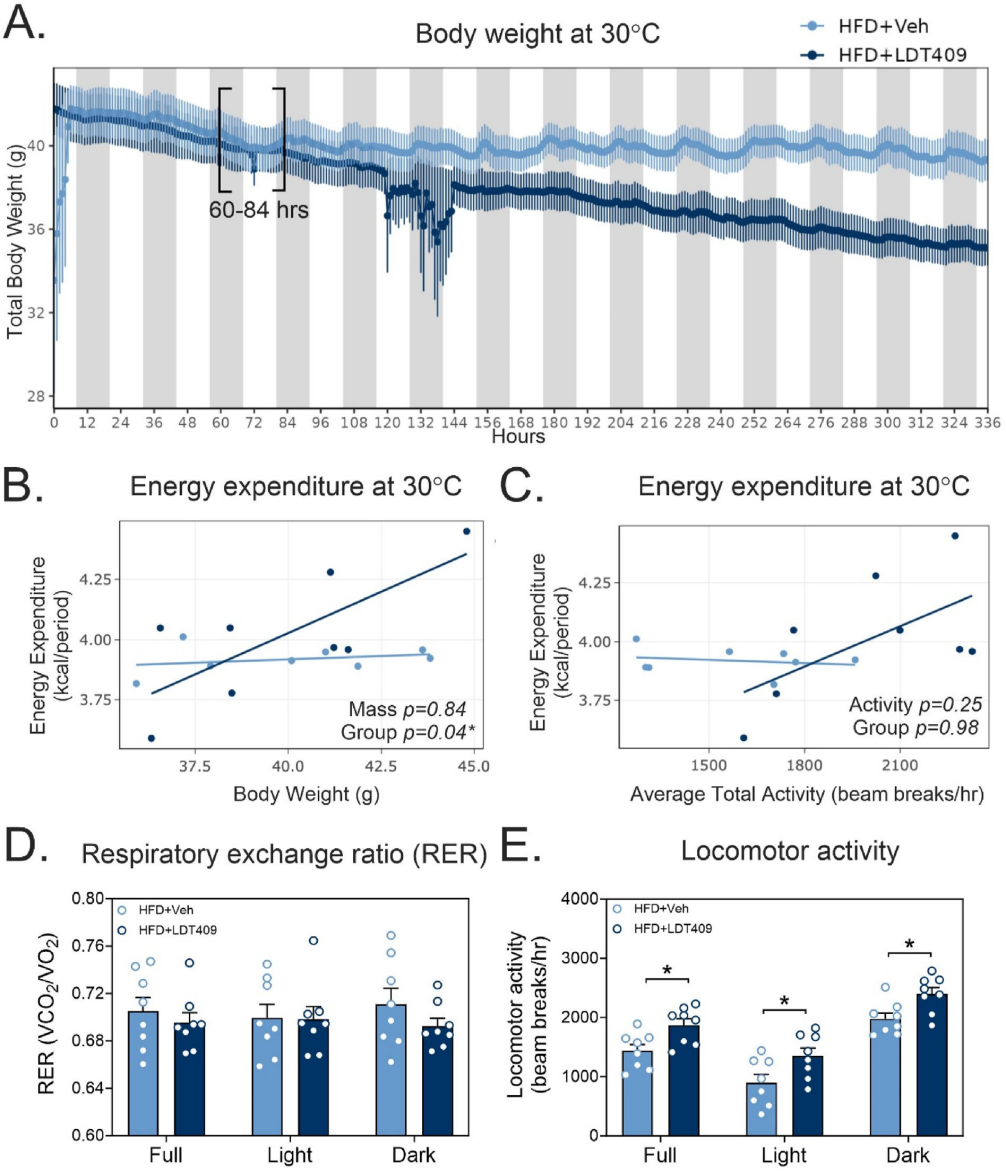
**LDT409 increases energy expenditure in HFD-fed mice prior to weight loss when housed at thermoneutrality.** (A–E) Mice with DIO were individually housed in metabolic cages at thermoneutrality (30 °C) and received vehicle or LDT409 treatment for 14 days. (A) Body weight of vehicle and LDT409-treated male mice fed a HFD for 14 days, n = 8 per group. (B) Regression plot of energy expenditure from HFD-fed mice treated with vehicle or 40 mg/kg LDT409 obtained from the indicated 24 h time slot (60–84 h of injections), n = 8. (C) Regression plot of energy expenditure versus total activity in mice. (D) Respiratory exchange ratio (RER) and (E) locomotor activity of HFD-fed mice received vehicle or 40 mg/kg LDT409 for 24 h, n = 8. (A, D, E) These data represent the average ± SEM. (B–C) Analyzed by ANCOVA using *CalR* with body weight or activity as a covariate. (D–E) ^∗^*P* < 0.05 vs indicated group using t-test.

Mice housed at room temperature (21–23 °C) undergo adaptive thermogenesis to maintain their core body temperature [72,73]. Therefore, to examine the effect of LDT409 on thermogenesis, HFD-fed mice were housed at thermoneutral condition (30 °C), where adaptive thermogenesis is minimally active. We found that LDT409-treated mice exhibited a significant decrease in body weight compared to vehicle-treated control mice at thermoneutrality (Figure 8A). Consistently, LDT409-treated mice ate less than HFD-vehicle mice and had a significant lower RER (Supplemental Figs. 11A and 11C). No significant differences were observed in daily locomotor activity or oxygen consumption (Supplemental Figs. 11B and 11D). We analyzed EE over a 24 h period (between 60 and 84 h) during which body weight and food intake were unchanged between treatment groups (Figure 8A, Supplemental Fig. 11A). Regression analyses of EE against mass and locomotor activity (Figure 8C,D) showed a positive correlation with LDT409 treatment vs mass that differed from vehicle treatment (Mass, *P* = 0.84; Group, *P* = 0.04) (Figure 8B). While RER levels were not changed at this point during treatment with LDT409, locomotor activity was remarkably increased in HFD-fed mice with LDT409 (Figure 8D,E). Together, these results indicate that LDT409 treatment initiates increased EE, which can later contribute to weight-loss in LDT409-treated mice.

## 4 Discussion

Herein, we investigated the molecular mechanisms governing the dramatic weight loss observed with the fatty acid mimetic LDT409 in a mouse model of diet-induced obesity. We report that LDT409 exhibited this potent anti-obesogenic effect in HFD-fed mice, but not chow-fed mice, with the weight loss that correlated with reduced fat mass. As expected, the dual PPARα/γ activation induced by LDT409 regulated browning of WAT, increased thermogenesis in brown adipocytes and enhanced glucose homeostasis. In addition, mechanistic studies demonstrate that LDT409 increased PPARα-targeted fatty acid oxidation, preventing lipid accumulation in the liver. Lastly, we found that LDT409 lowered both food intake and intestinal energy absorption. The overall effect of this multi-targeted small molecule was a very effective weight-loss treatment that also improved dyslipidemia and glucose control.

PPARα agonists (e.g., OEA and fenofibrate) have been shown to ameliorate dyslipidemia and decrease body weight in rodents [33,74]. Specifically, fenofibrate protects against Rosi-induced weight gain in *ob/ob* mice [75], implying that dual PPARα/γ agonism could counteract PPARγ-induced weight gain. However, unexpectedly, dual PPARα/γ agonists such as muraglitazar and tesaglitazar, increased body weight in rodents [76], likely due to the higher selectivity of PPARγ from these dual PPARα/γ agonists [31]. In our study, we observed that LDT409 reversed DIO, in line with its more balanced activity toward PPARα and PPARγ. Notably, LDT409 promoted weight loss in DIO mice, independent of sex, and did not influence body weight in lean mice, suggesting it selectively promotes fat utilization. We also observed that LDT409 induced anorectic effects in obese mice, in line with decreased food consumption previously observed in OEA-treated rats, which was attributed to the stimulation of the paraventricular and supraoptic nuclei in the hypothalamus [33,77]. This raised the question of whether the decreased food intake with LDT409 could account fully for the weight-loss we observed. The pair-feeding study revealed that lower food intake was not able to fully account for reduction in body weight observed in LDT409-treated obese mice.

FGF21, a liver-secreted endocrine hormone, is a direct target of PPARα [51,78]. Previous studies show that mice overexpressing FGF21 are resistant to HFD-induced obesity [79] while whole-body deletion of FGF21 results in slight weight gain and impaired blood glucose control compared to WT mice [80]. Additionally, primates administered pharmacologic doses of recombinant FGF21 (rFGF21) demonstrated weight loss without a change in food intake [81,82]. Weight loss was also observed for doses of rFGF21 administered to obese mice yielding plasma concentrations of >60 ng/mL [[83], [84], [85]]. However, when lower doses of rFGF21 were administrated to obese mice (yielding plasma concentrations between 7 and 13 ng/mL), no changes to body weight, adiposity, or EE were observed [53,84,85]. The levels of circulating FGF21 produced following WY or LDT409-treatment (plasma concentration ∼1.4 ng/mL) are too low to stimulate FGF21-dependent weight loss and EE. Likewise, because treatment with WY increased FGF21 levels to the same extent as LDT409 but did not cause significant weight loss, we do not think FGF21 is a primary mediator of our weight loss phenotype.

Activation of BAT plays a potential role in the treatment of obesity and diabetes [66,86]. Recent studies have shown that activation of brown adipocytes enhances substrate oxidation (e.g., glucose and fat oxidation) in humans, contributing to improvement of cardiometabolic health [66,87]. Our histology and gene expression data suggest LDT409 decreased lipid storage and increased thermogenesis in adipose tissue. This is consistent with PPARγ-induced browning in WAT and thermogenesis in BAT [27,58]. These factors would be expected to promote weight loss and improve glucose homeostasis. Consistent with increased thermogenic gene expression, LDT409 treatment of T37i brown adipocytes, increased oxygen consumption which was attributed to increased proton leak and uncoupled respiration. However, when we measured EE by indirect calorimetry, HFD-fed mice treated with LDT409 showed no significant difference from vehicle at room temperature. It is possible that the indirect calorimetry system was not sufficiently sensitive to detect changes in EE under these conditions [88,89]. When the study was repeated at thermoneutrality and analyzed at a timepoint prior to weight loss, LDT409 did significantly enhance EE. LDT409 also decreased RER values (suggesting enhanced fat utilization) and decreased food intake, resulting in a negative energy balance that would also contribute to the weight loss observed.

We observed increased liver weight and larger intrahepatic lipid droplets in mice treated with Rosi compared to vehicle, phenotypes that have previously been attributed to increased hepatosteatosis and upregulation of fat droplet-associated genes (*Cidec* and *Pparγ2)*, respectively [54,55,90,91]. In contrast, these PPARγ-induced side effects were not observed in LDT409-treated mice, suggesting that LDT409 preferentially agonizes PPARα over PPARγ in the liver.

PPARα agonists have been shown to decrease hepatic steatosis by promoting both the uptake and oxidation of fat in the liver [33,74,[92], [93], [94], [95]]. As expected, LDT409 and WY significantly decreased liver and plasma triglyceride levels while also upregulating hepatic fatty acid uptake and oxidation genes. Interestingly, LDT409 differentially impacted plasma and liver cholesterol compared to vehicle. LDT409 reduced both plasma and liver cholesterol while WY had no effect on liver cholesterol and increased total plasma cholesterol compared to HFD controls. The effect of WY to increase plasma cholesterol may be related to its ability to increase the secretion of ApoB-100 which promotes the formation of IDL/LDL via increased *Mttp* (microsomal triglyceride transfer protein) expression [49,96]. Thus, LDT409 confers a uniquely beneficial lipid and cholesterol profile in plasma and liver, distinct from the known anti-lipidemic activity of the PPARα agonist WY. This raises the possibility that LDT409 acts independently of hepatic PPARα to impact cholesterol homeostasis.

We considered the importance of the intestinal epithelium as another site of action due to its essential role in lipid uptake and lipid oxidation. Studies have shown that PPARs increase fatty acid oxidation in enterocytes [97] while deletion of intestinal PPARα reduces lipid absorption and synthesis in the small intestine [98]. Consistent with previous literature reporting highly efficient absorption of lipids from the intestine [99], we found that dietary lipid was very efficiently absorbed (∼99%) in mice fed a HFD but this was blunted in mice treated with LDT409 (to 97.5%). Pharmacological activation of PPARα or PPARδ suppresses intestinal cholesterol absorption through downregulation of Niemann-Pick C1-Like 1 (NPC1L1) [100,101]. Consistent with these findings, LDT409 tended to decrease the mRNA expression of *Npc1l1* and significantly increased the excretion of cholesterol into feces. Therefore, we show that LDT409 inhibited intestinal lipid absorption, which could contribute to the decrease in circulating triglycerides and cholesterol and contribute to weight loss over time.

Exposure to a HFD leads to adipose tissue inflammation, increasing the risk for metabolic dysregulation [102]. Epidydimal WAT, in contrast to inguinal or mesenteric WAT, is more prone to develop chronic inflammation [103]. Previous studies have shown that Rosi exhibits anti-inflammatory effects to inhibit the expression of cytokine-related genes in WAT [94,[104], [105], [106]]. PPARα agonists also suppress inflammation through inactivation of NF-kB and have been shown to reduce M1-macrophages in adipose tissue of diabetic KKAy mice [[107], [108], [109]]. In our studies, we found that LDT409 reduced the induction of inflammatory genes in LPS-stimulated RAW264.7 macrophage cells as well as in eWAT of HFD-fed mice. These findings suggest that LDT409 may potentiate greater anti-inflammatory effects through combined activation of PPARα and PPARγ.

## 5 Conclusion

HFD feeding promotes obesity, disturbs the metabolic flexibility from glucose oxidation to fatty acid oxidation that leads to insulin resistance, and increases fat accumulation in non-adipose tissues. LDT409 has a beneficial effect on decreasing food intake and hyperlipidemia, while ameliorating hepatic steatosis and enhancing insulin tolerance. Compared to vehicle-treated mice, mice treated with LDT409 were resistant to HFD-induced adipocyte hypertrophy, exhibited browning of their WAT depots, showed evidence of increased whole-body fat oxidation and reduced inflammation in WAT when compared with vehicle-treated mice on HFD. Additionally, LDT409 normalized the HFD-induced weight gain back to chow-fed control mice. Mechanisms contributing to the reduction in body weight when mice received LDT409 treatment include a decrease in food intake, an increase in EE and fatty acid utilization, and a minor decrease in intestinal absorption of nutrients. Our results collectively suggest that LDT409, with its structural similarly to endogenous fatty acids, provides a unique tissue optimized activity profile for PPARα and PPARγ that could be beneficial to treat a variety of metabolic diseases including obesity, diabetes and MASLD. Given the abundant global supply of starting material for LDT409, it could serve as a low-cost therapeutic for resource-limited countries that are also suffering from the rising global burden of metabolic disease.

## CRediT authorship contribution statement

**Cigdem Sahin:** Writing – original draft, Visualization, Methodology, Investigation, Formal analysis, Data curation, Conceptualization. **Jenna-Rose Melanson:** Visualization, Investigation, Formal Analysis, Methodology. **Florian Le Billan:** Writing - review & editing, Investigation. **Lilia Magomedova:** Investigation, Methodology. **Thais A.M. Ferreira:** Resources, Investigation. **Andressa S. Oliveira:** Resources, Investigation. **Evan Pollock-Tahari:** Investigation. **Michael F. Saikali:** Writing – review & editing, Visualization, Formal analysis, Data curation, Investigation **Sarah B. Cash:** Writing - review & editing, Investigation. **Minna Woo:** Writing – review & editing, Supervision, Resources. **Luiz A.S. Romeiro:** Writing – review & editing, Supervision, Resources, Funding acquisition, Conceptualization. **Carolyn L. Cummins:** Writing – review & editing, Supervision, Resources, Project administration, Funding acquisition, Formal analysis, Conceptualization, Methodology.

## Declaration of competing interest

CLC, LASR, and LM are co-authors on a patent related to this work. They wish to pursue collaborations to further develop LDT409 as an orally bioavailable low-cost therapy to treat obesity-related metabolic disease.

## Data Availability

Data will be made available on request.
