# Supplementary material for: A novel fatty acid mimetic with pan-PPAR partial agonist activity inhibits diet-induced obesity and metabolic dysfunction-associated steatotic liver disease
Source: Mol Metab. 2024 May 17;85:101958. doi: 10.1016/j.molmet.2024.101958 (PMC11170206; doi:10.1016/j.molmet.2024.101958)
Supplement: Multimedia component 1 [file mmc1.docx]

Supplementary Data

A Novel Fatty Acid Mimetic with Pan-PPAR Partial Agonist Activity Inhibits Diet-Induced Obesity and Metabolic Dysfunction-Associated Steatotic Liver Disease

Cigdem Sahin^1^, Jenna-Rose Melanson^1^, Florian Le Billan^1^, Lilia Magomedova^1^, Thais A. M. Ferreira^2^, Andressa S. Oliveira^2^, Evan Pollock-Tahari^3^, Michael F. Saikali^1^, Sarah B. Cash^1^, Minna Woo^3,4^, Luiz A. S. Romeiro^2^, Carolyn L. Cummins^1,4*^

^1^Department of Pharmaceutical Sciences, Leslie Dan Faculty of Pharmacy, University of Toronto, Toronto, ON M5S 3M2, Canada

^2^Department of Pharmacy, Faculty of Health Sciences, University of Brasilia, Brasilia, DF 71910-900, Brazil

^3^Toronto General Hospital Research Institute, University Health Network, Toronto, ON, M5G 2C4, Canada

^4^Banting and Best Diabetes Centre, Toronto, ON, M5G 2C4, Canada


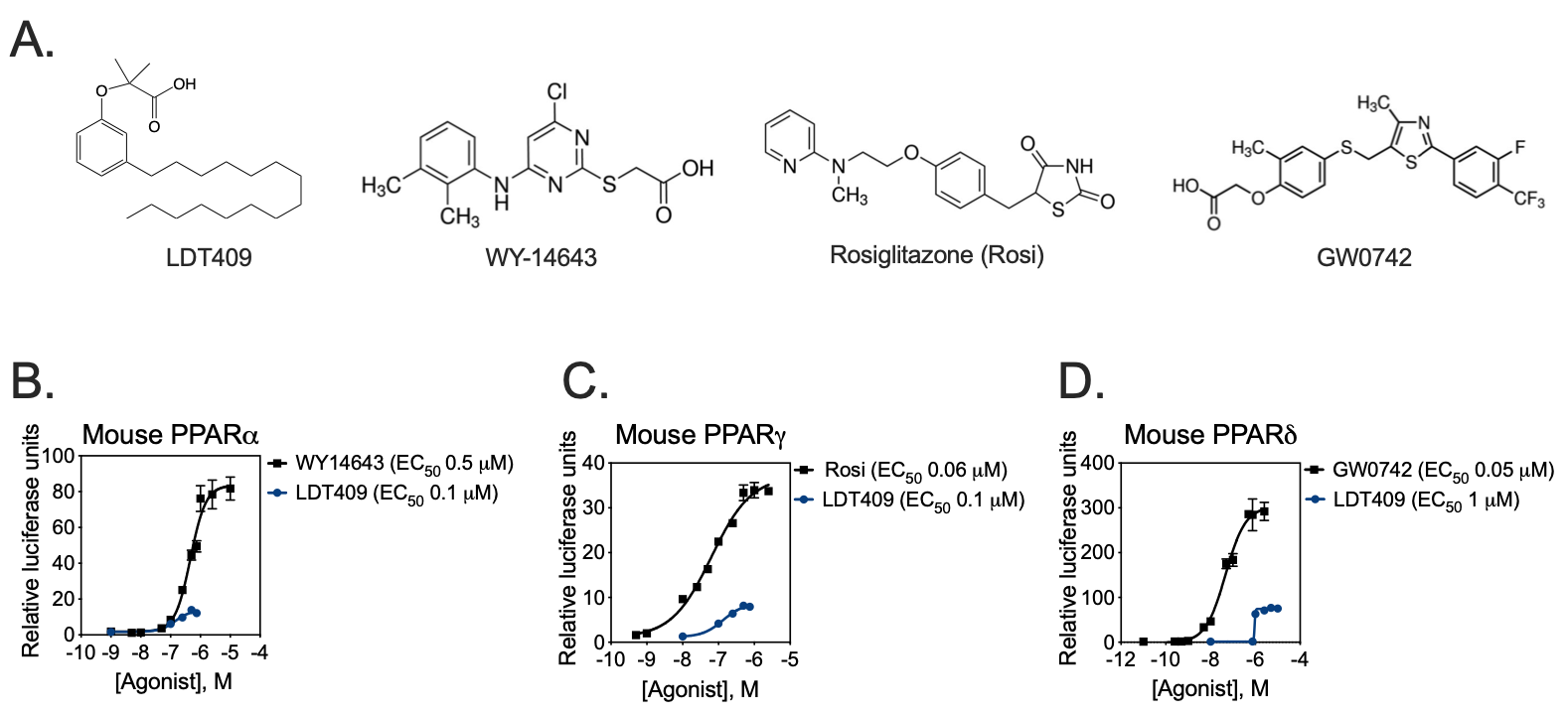


Supplemental Figure 1. LDT409 is a partial agonist of mouse PPARα, PPARγ, and PPARδ *in vitro*.

(**A**) Chemical structure of LDT409 (pan-PPARα/γ/δ agonist), WY14643 (WY, PPARα agonist), Rosiglitazone (Rosi, PPARγ agonist), and GW0742 (GW, PPARδ agonist). (**B-C**) HEK293 cells were co-transfected with mouse GAL4-PPARα, GAL4-PPARγ, or GAL4-PPARδ, UAS-luciferase reporter and β-galactosidase plasmid. Six hours post-transfection, cells were treated with increasing concentrations of LDT409, WY, Rosi, or GW and harvested for luciferase assay 16 h later. To control for transfection efficiency, luciferase values were normalized to β-galactosidase. Data show a representative experiment mean ± SD (N=3). Relative luciferase units = luciferase light units/β-galactosidase*time. Vehicle (DMSO) response was set to 1. EC_50_ values determined using nonlinear regression with a variable slope (four parameters) curve fit (Prism, GraphPad).


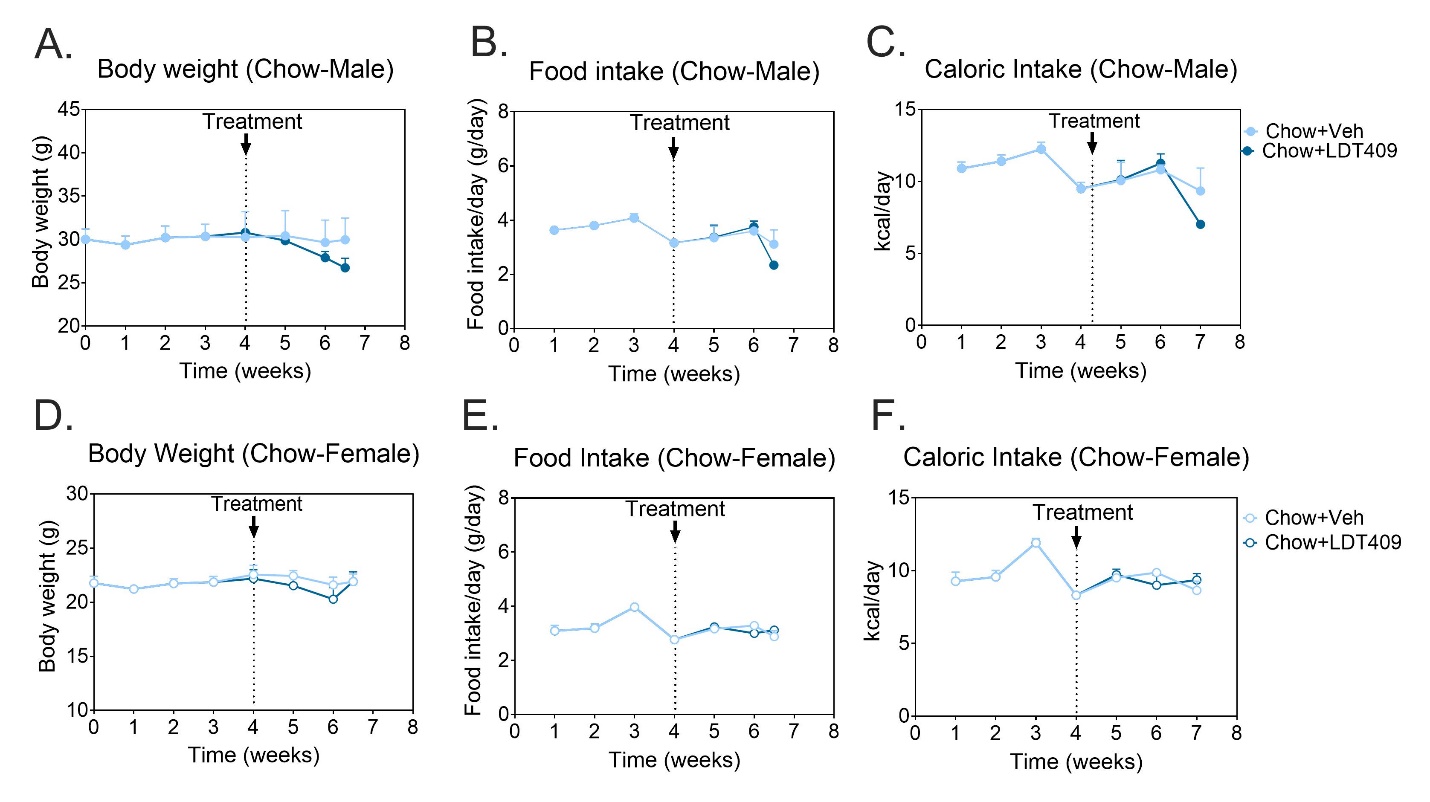


Supplemental Figure 2. LDT409 has no impact on body weight and food intake in chow-fed mice.

(**A-F**) Chow-fed mice received vehicle or LDT409 for 2.5 weeks (18 days). (**A**) Body weight of male chow-fed mice. (**B**) Daily food intake of male mice. (**C**) Caloric intake of male mice. Caloric intake (kcal/day) = Food intake (g diet/day) x caloric value of diet (kcal/g). (**D**) Body weight of female chow-fed mice. (**E**) Daily food intake of female mice. (**F**) Caloric intake of female mice. Data represent the average ± SEM, n=3-4. **P*<0.05 vs Chow+Veh, using two-way ANOVA with Holm-Sidak correction. Chow+Veh: Chow-fed mice treated with vehicle; Chow+LDT409: Chow-fed mice treated with LDT409.


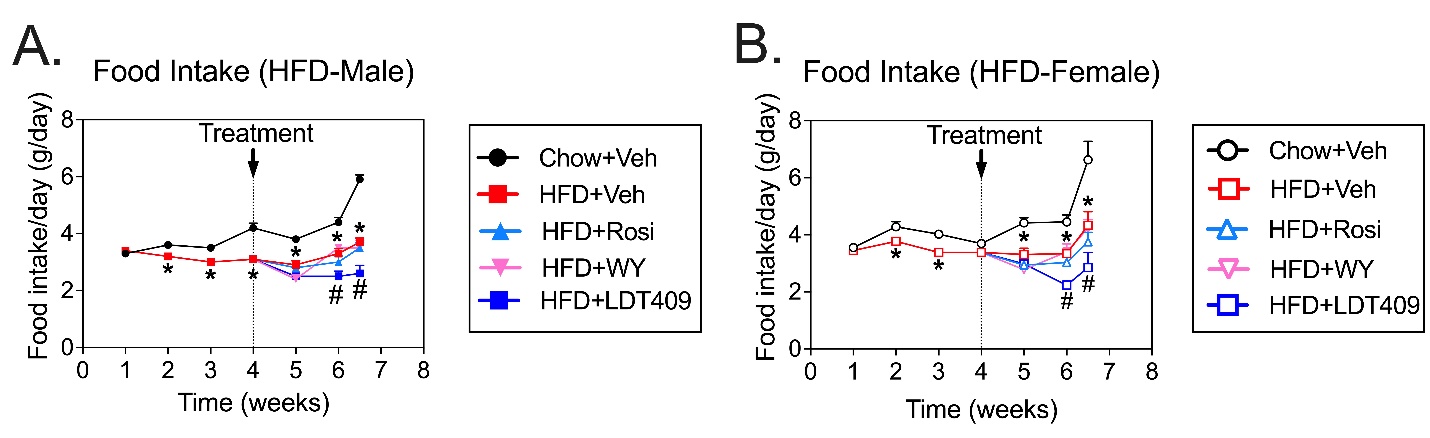


Supplemental Figure 3. Food intake is decreased in LDT409 treated HFD-fed male and female mice.

(**A-B**) Mice with DIO received vehicle, rosiglitazone (Rosi), WY14643 (WY) or LDT409 for 2.5 weeks (18 days). (**A**) Food intake of male mice, n=6-8 per group. (**B**) Food intake of female mice, n=4-5 per group. Data represent the average ± SEM. **P*<0.05 vs Chow+Veh, ^#^*P*<0.05 vs HFD+Veh using two-way ANOVA with Holm-Sidak correction.


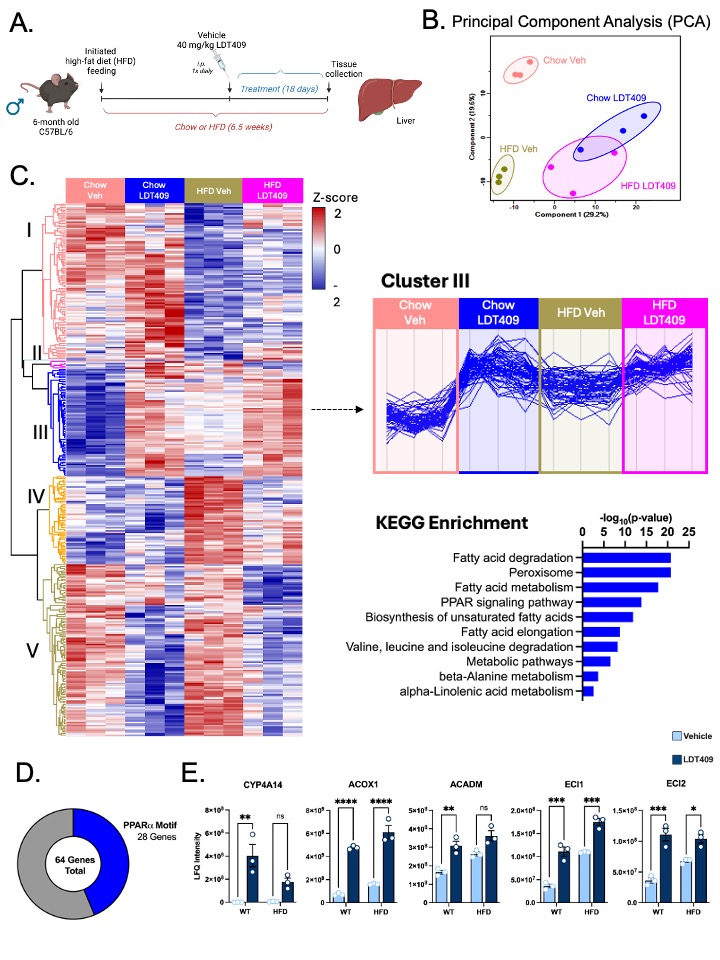


Supplemental Figure 4. LDT409-induced changes in the liver proteome.

(**A**) Schematic of experimental setup. Liver samples were obtained from Chow-Veh; Chow-LDT409; HFD-Veh or HFD-LDT409 6-month-old male mice, n=3 per group. (**B**) Principal component analysis of the liver proteome showing distinct clusters. (**C**) Heatmap of differentially expressed proteins in chow or high fat diet fed mice treated with LDT409. The rows (representing individual proteins) clustered into 5 groups for further analysis. A profile plot of cluster III is shown to highlight the trends in overall expression. The top 10 enriched KEGG terms for cluster III are plotted. (**D**) Cluster III genes were analyzed by TRANSFAC and 28 of the queried proteins showed enrichment for a PPARα motif in their gene promoters (**E**) Representative plots of individual proteins. CYP4A14, ACOX1, ACADM, ECI1, and ECI2 are known PPARα target genes involved in fatty acid oxidation. Data shown are the average ± SEM n=3, **P*<0.05, ***P*<0.01, ****P*<0.001, *****P*<0.0001 by two-way ANOVA with Holm-Sidak correction.


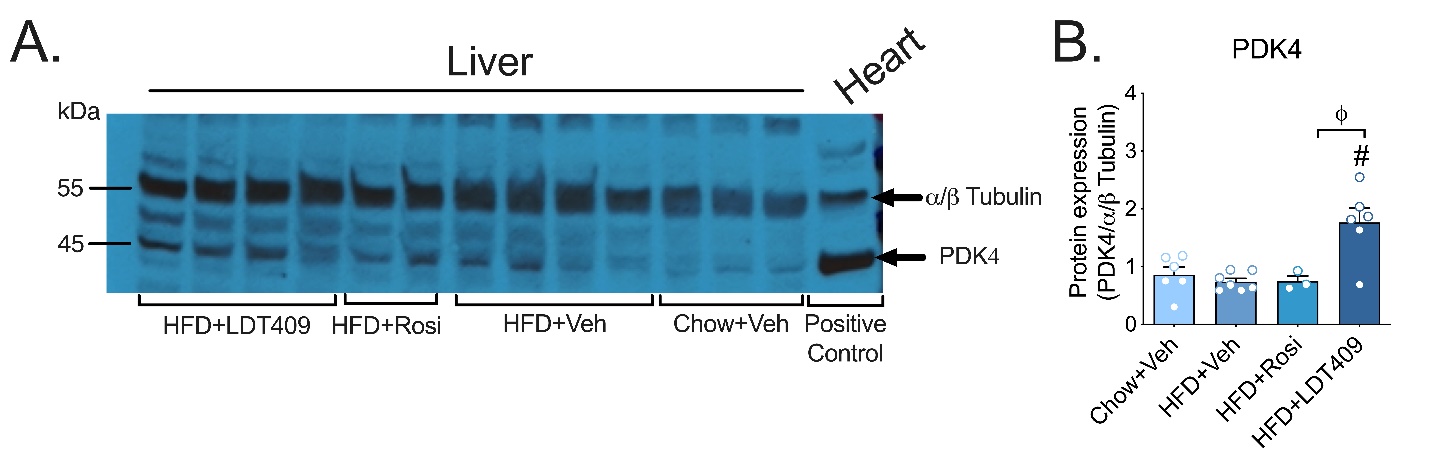


Supplemental Figure 5. LDT409 increases PDK4 protein in livers of HFD-fed male mice.

(**A-B**) Mice with DIO received vehicle, rosiglitazone (Rosi) or LDT409 for 2.5 weeks (18 days).

(**A**) Representative immunoblot showing PDK4 protein in liver homogenates with α/β Tubulin as a loading control. (**B**) Quantification of PDK4 protein relative to α/β Tubulin in the liver, n=3-6. Data represent the average ± SEM (**A-B**, n=3-6). **P*<0.05 vs Chow+Veh, #*P*<0.05 vs HFD+Veh, ^φ^*P*<0.05 vs indicated group using one-way ANOVA with Holm-Sidak correction.

Supplemental Figure 6. LDT409 reduces LPS-induced inflammation in RAW264.7 cells.

Quantitative real-time PCR analysis for expression of pro-inflammatory marker genes (*Tnfα*, *Il-1β*, and *Mcp1*) in RAW264.7 macrophage-like cells was completed. RAW264.7 cells were treated with vehicle or 25 μM LDT409 for 24 h and then, cells were treated with no LPS, 10 ng/mL LPS or 10 ng/mL LPS+ 25 μM LDT409 for additional 6 h. These data represent the average ± SEM. **P*<0.05 vs indicated group using one-way ANOVA with Holm-Sidak correction.

Supplemental Figure 7. LDT409 increases fatty acid oxidation gene expression in differentiated T37i brown adipocyte cells.

Quantitative real-time PCR analysis of genes implicated in fatty acid oxidation in differentiated T37i brown adipocytes in the absence or presence of 25 μM LDT409 for 16 h, n=3-4.

These data represent the average ± SEM. **P*<0.05 vs Vehicle using a t-test.


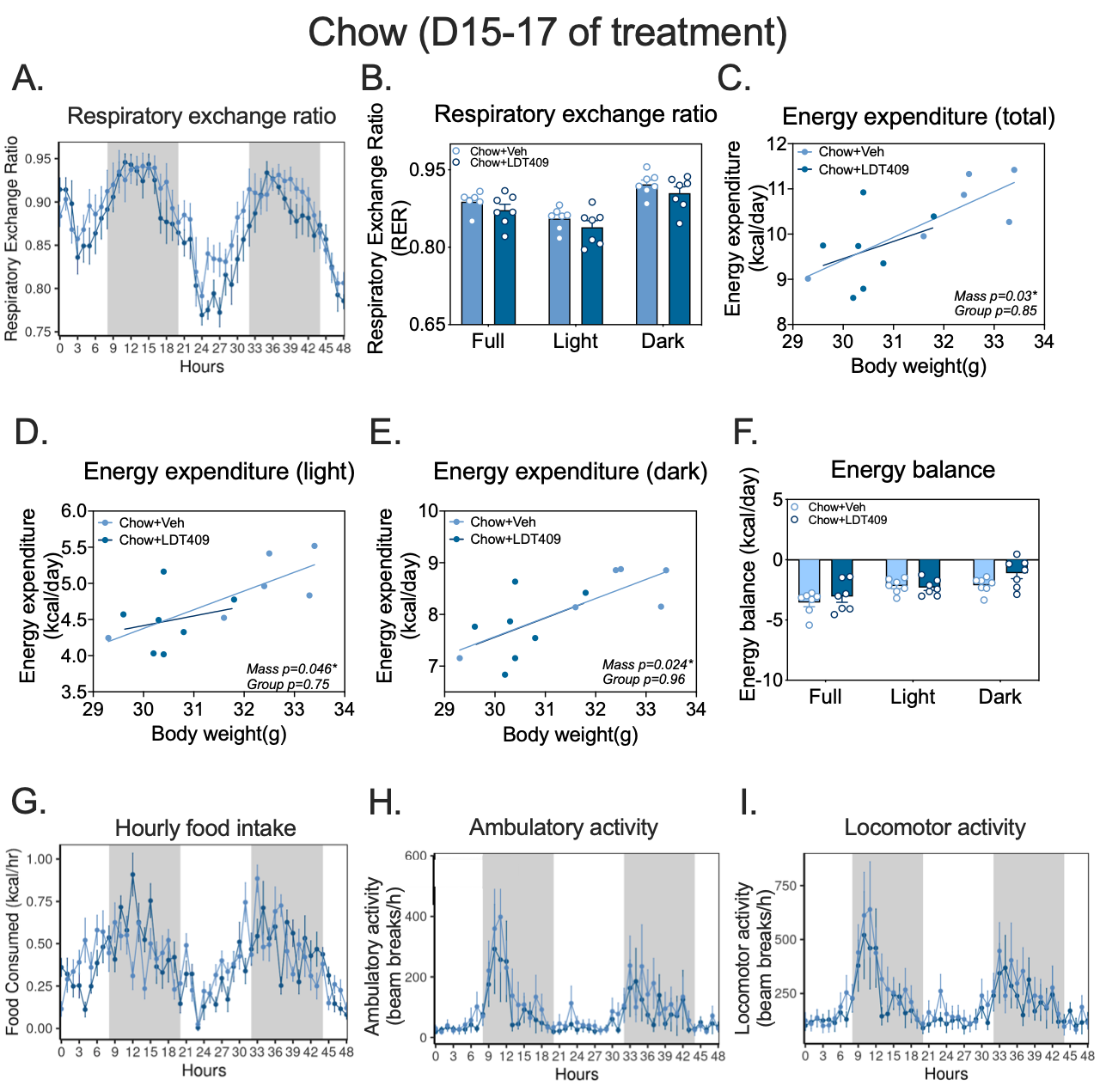


Supplemental Figure 8. LDT409 has no impact on energy metabolism in chow-fed mice housed at room temperature.

(**A-I**) Male mice were treated with vehicle or LDT409 for 17 days and housed at room temperature. (**A-B**) Respiratory exchange ratio (RER) of chow-fed mice during the final 48 h of vehicle or 40 mg/kg LDT409 treatment. (**C-E**) Regression plot of energy expenditure from total (**C**), light (**D**), and dark cycles (**E**), in the final 48 h of vehicle or 40 mg/kg LDT409 treatment. (**F**) Energy balance in last 48 h of chow-fed mice receiving vehicle or 40 mg/kg LDT409. (**G**) Hourly food intake in last 48 h as kcal. (**H-I**) Ambulatory and locomotor activity profiles in last 48 h from chow-fed mice treated with vehicle or 40 mg/kg LDT409. *CalR* was used to analyze these data as described by Mina et al., 2018 [1]. Data shown are the average ± SEM, n=6-7.


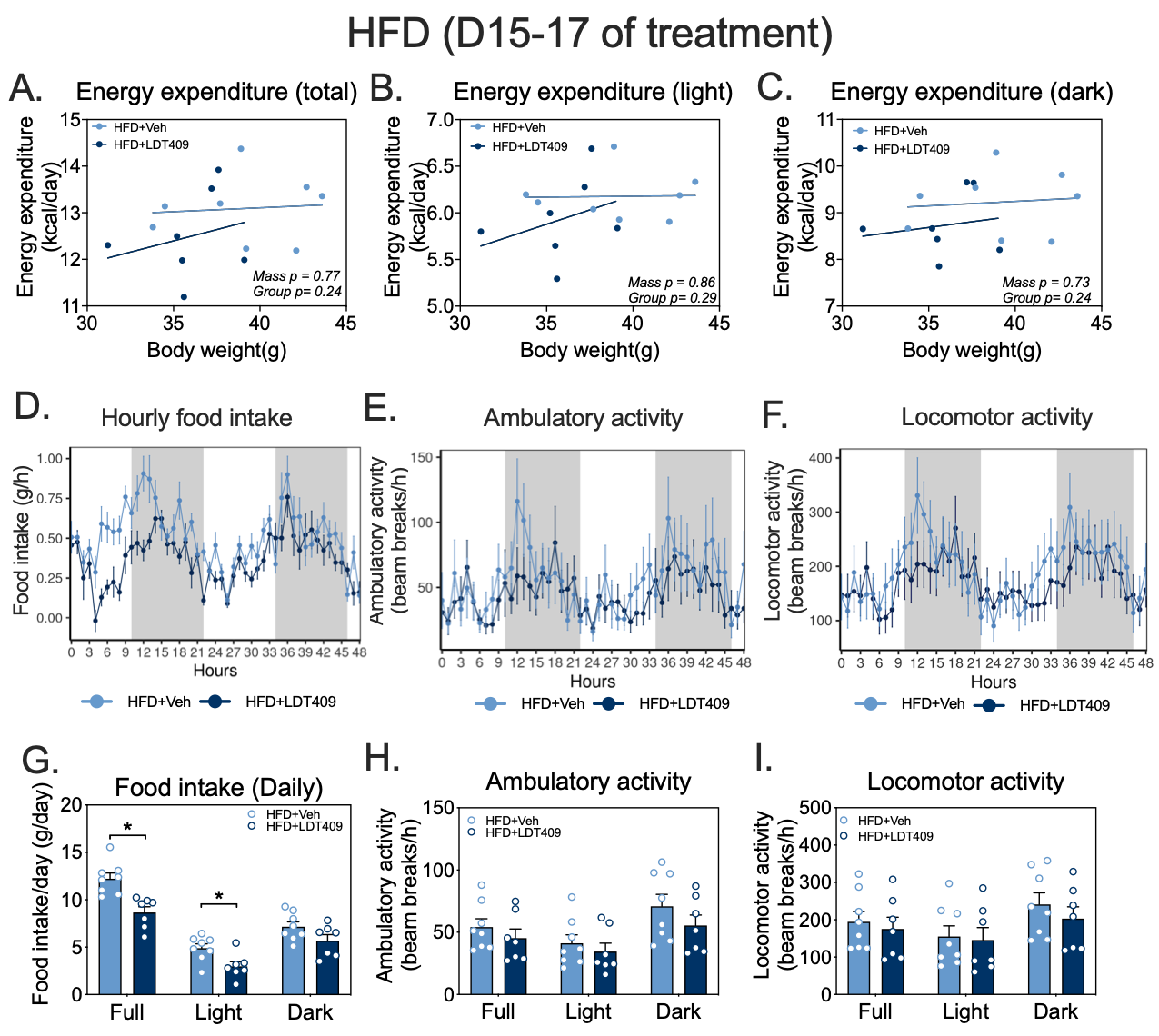


Supplemental Figure 9. Effect of LDT409 on metabolic profiles of HFD-fed mice housed at room temperature.

(**A-I**) Male mice with DIO received vehicle or LDT409 treatment for 17 days. (**A-C**) Regression plot of energy expenditure vs body weigh during full, light, and dark cycles. (**D**) Hourly food intake for 48 h. (**E-F**) Ambulatory and locomotor activity profiles from HFD-fed mice with vehicle or 40 mg/kg LDT409. (**G**) Daily average food intake for 48 h. (**H-I**) Daily ambulatory and locomotor activity. *CalR* was used to analyze these data as described by Mina et al., 2018 [1]. Data shown are the average ± SEM, n=7-8. **P*<0.05 vs indicated group.


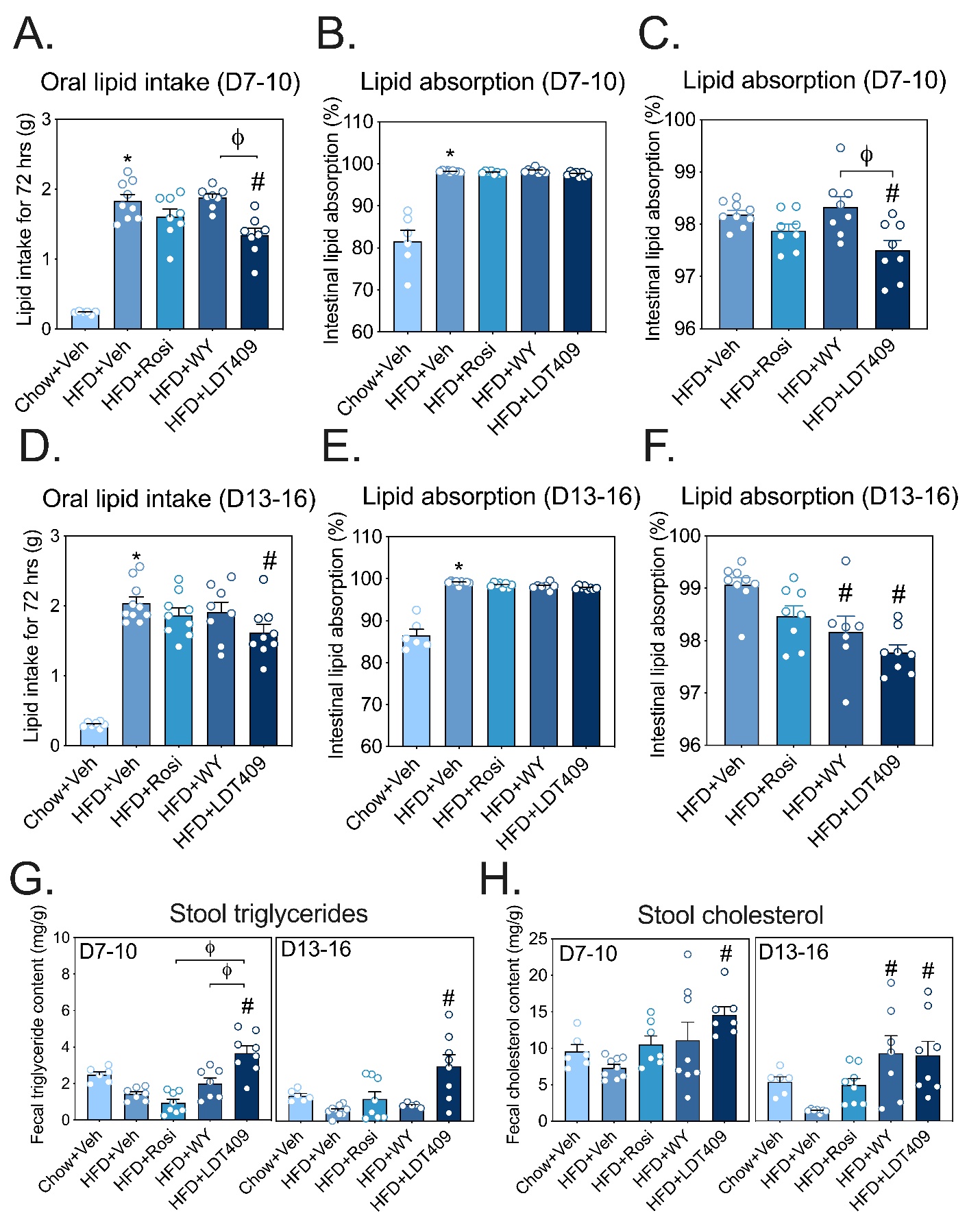


Supplemental Figure 10. LDT409 limits intestinal lipid absorption in DIO mice.

Lean male mice were treated with vehicle, whereas HFD-fed obese mice were treated with vehicle, 10 mg/kg Rosi, 40 mg/kg WY, or 40 mg/kg LDT409 treatment for 18 days. Between day 7 and 10 of treatment, (**A**) oral lipid intake from the diet was monitored for 72 h and (**B**) feces were collected to measure lipid absorption for 72 h. (**C**) Data re-plotted from panel (**B**), excluding chow fed control. Between day 13 and 16 of treatment, (**D**) oral lipid intake from the diet was monitored for 72 h and feces were collected (**E-F**). Lipid absorption with (**E**) or without (**F**) chow-fed mice were determined for 72 h. (**G-H**) Excretion levels of stool triglyceride and stool cholesterol. Data represent the average ± SEM, n=6-9 per group. **P*<0.05 vs Chow+Veh, ^#^*P*<0.05 vs HFD+Veh, ^φ^*P*<0.05 vs indicated group using one-way ANOVA with Holm-Sidak correction.


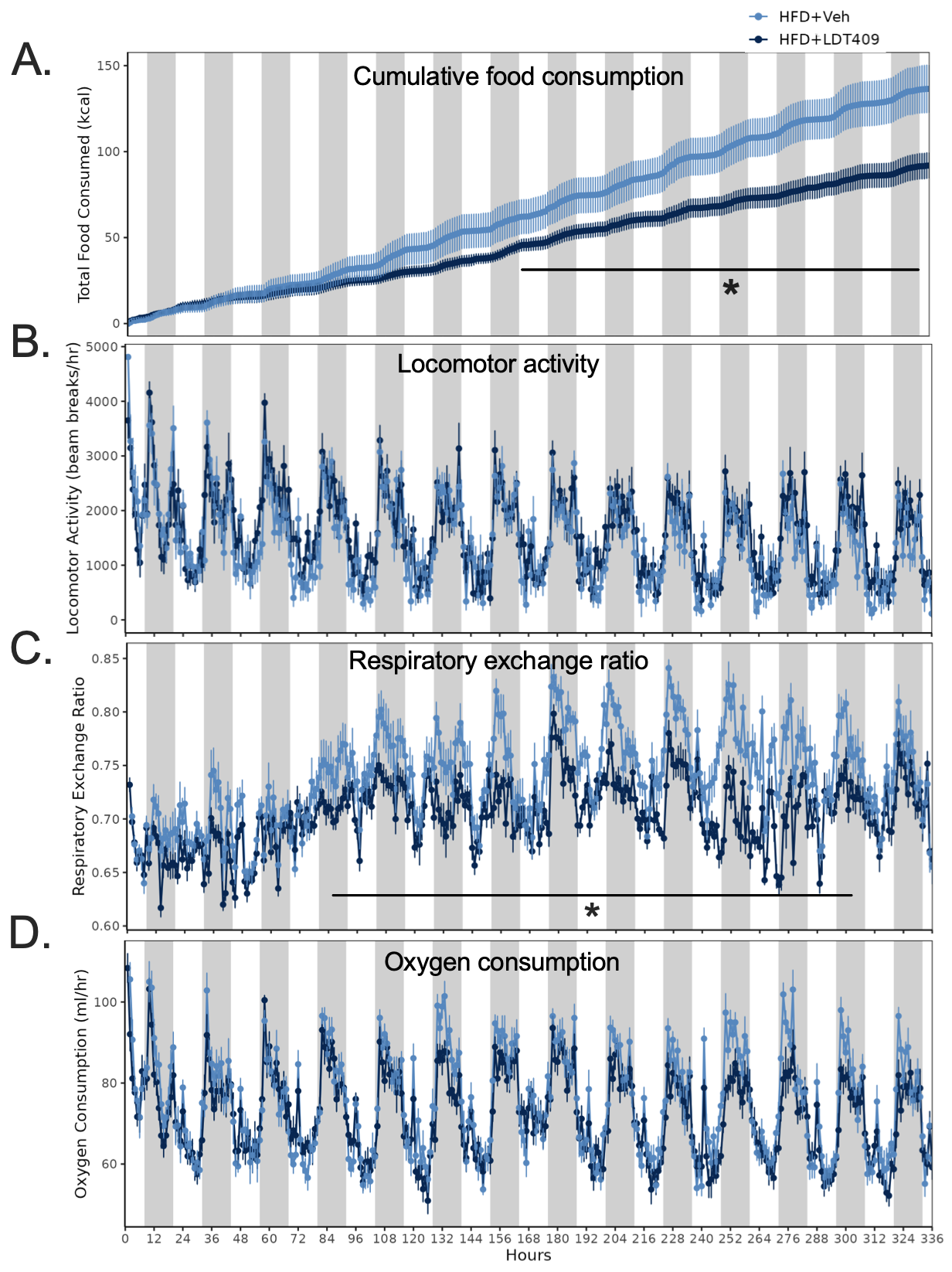


Supplemental Figure 11. LDT409 treatment influences metabolic outcomes in HFD-fed mice housed at thermoneutrality.

Male mice with DIO were individually housed in metabolic cages at thermoneutrality (30 °C) and received vehicle or 40 mg/kg LDT409 treatment for 14 days. (**A**) Cumulative food consumption, (**B**) locomotor activity, (**C**) respiratory exchange ratio, and (**D**) oxygen consumption of HFD-fed male mice treated with Vehicle or LDT409 for 14 days. These data represent the average ± SEM, n=8 per group. **P*<0.05 vs indicated group.

Supplementary Table S1. Mouse QPCR primer sequences

| **Gene Name** | **Abbrev.** | **Accession No.** | **Forward and Reverse Primer Sequences (5’-3’)** |
| --- | --- | --- | --- |
| Acidic ribosomal phosphoprotein P0 | *36b4* | NM_007475.5 | cgtcctcgttggagtgaca  cggtgcgtcagggattg |
| Acetyl-CoA carboxylase alpha | *Acaca* | NM_133360.2 | ggacagactgatcgcagagaaag  tggagagccccacacaca |
| Acyl-Coenzyme a dehydrogenase | *Acadl* | NM_007381.4 | tctgggagtgattggattctca  acgagatcacttaaccagccattagt |
| Alkaline phosphatase, biomineralization associated | *Alpl* | NM_007431.3 | tcaacaccaatgtagccaaga  gtagctggcccttaaggattc |
| Cluster of differentiation 36 | *Cd36* | NM_007643.3 | gaactgtgggctcattgc  catgagaatgcctccaaacac |
| Cluster of differentiation 68 | *Cd68* | NM_009853.1 | tggcggtggaatacaatgtg  gatgaattctgcgccatgaa |
| Cell death inducing DFFA like effector a | *Cidea* | NM_007702.2 | gccgtgttaaggaatctgctg  tgctcttctgtatcgcccagt |
| Cell death inducing DFFA like effector c | *Cidec/Fsp27* | NM_178373.3 | ggcaaaagataccatgttcatg  gcttctgggaaagggctagct |
| Carbohydrate response element binding protein | *Chrebp* | NM_021455.5 | ggacaagatccggctgaaca  ggctcttcctccgttgca |
| Cytochrome c oxidase subunit 7a | *Cox7a* | NM_009944.3 | cagcgtcatggtcagtctgt  agaaaaccgtgtggcagaga |
| Cytochrome c oxidase subunit 8b | *Cox8b* | NM_007751.3 | gaaaccatgaagccaacgact  gcgaagttcacagtggttcc |
| Carnitine palmitoyltransferase1 | *Cpt1* | NM_013495.2 | gcagagcacggcaaaatga  ctttcgacccgagaagacctt |
| Creatine kinase b | *Ckb* | NM_021273.4 | gcctcactcagatcgaaactc  ggcatgtgaggatgtagccc |
| Cyclophilin b | *Cyclophilin* | NM_011149.2 | ggagatggcacaggaggaa  gcccgtagtgcttcagctt |
| Diacylglycerol O-Acyltransferase 1 | *Dgat1* | NM_010046.2 | cgtgggcgacggctact  tgagctgaacaaagaatcttgca |
| Diacylglycerol O-Acyltransferase 2 | *Dgat2* | NM_026384.3 | gctggcatttgactggaaca  gccacacggcccagttt |
| Elongation of very long chain fatty acids protein 3 | *Elovl3* | NM_007703.2 | atgaatttctcacgcgggtt  agcttacccagtactcctcca |
| Fatty acid binding protein 1 | *Fabp1* | NM_017399.4 | agccattcatgaaggcaatagg  cccttgatgtccttccctttc |
| Fatty acid binding protein 2 | *Fabp2* | NM_007980.3 | tcctccaagatgtgcggtact  taggtgagcgtctcgtctcg |
| Fatty acid binding protein 4 | *Fabp4* | NM_024406.2 | ccgcagacgacaggaaggt  agggccccgccatct |
| Fatty acid synthase | *Fasn* | NM_007988.3 | gctgcggaaacttcaggaaat  agagacgtgtcactcctggactt |
| Fibroblast growth  factor 21 | *Fgf21* | NM_020013.4 | cctctaggtttctttgccaacag  aagctgcaggcctcaggat |
| G-protein coupled receptor 3 | *Gpr3* | NM_008154.3 | atcacctgagcaaccgagaa  agatgggggtgcattttaca |
| Interleukin 1 beta | *Il-1β* | NM_008361 | agttgacggaccccaaaaga  ggacagcccaggtcaaagg |
| Lipoprotein lipase | *Lpl* | NM_008509.2 | ggccagattcatcaactggat  gctccaaggctgtaccctaag |
| Monocyte chemoattractant protein 1 | *Mcp1* | NM_011333.3 | caggtgtcccaaagaagctgtag  gggtcagcacagacctctctct |
| Niemann-Pick C1-Like 1 | *Npc1l1* | NM_207242.2 | gcaaggtgatcaggaggttga  atcctcatcctgggctttgc |
| Phosphoenolpyruvate carboxykinase | *Pck1* | NM_011044.2 | caccatcacctcctggaaga  gggtgcagaatctcgagttg |
| Pyruvate dehydrogenase lipoamide kinase isozyme 4 | *Pdk4* | NM_013743.2 | ggttttacatgaaccgcatttcta  tttcccgtctttgagtcactga |
| PPAR-gamma coactivator 1 alpha | *Ppargc1α/*  *Pgc1α* | NM_008904.1 | accacacccacaggatcagaa  tcttcgctttattgctccatga |
| PR domain containing 16 | *Prdm16* | NM_001177995.1 | cgacaccatgcgatccaa  ccgggtcaggttcatacatattatt |
| Peroxisome proliferator-activated receptor gamma 2 | *Pparγ2* | NM_011146.3 | tcgctgatgcactgcctatg  gagaggtccacagagctgatt |
| Stearoyl-CoA desaturase 1 | *Scd-1* | NM_009127.3 | tgcccctgcggatctt  gcccattcgtacacgtcatt |
| Sterol regulatory element-binding transcription factor 1c | *Srebp1c* | NM_011480.2 | ggagccatggattgcacatt  ggcccgggaagtcactgt |
| Tissue-nonspecific alkaline phosphatase | *Alpl* | NM_007431.3 | tcaacaccaatgtagccaaga  gtagctggcccttaaggattc |
| Tumor necrosis factor alpha | *Tnfα* | NM_013693.2 | ctgaggtcaatctgcccaagtac  cttcacagagcaatgactccaaag |
| Uncoupling protein 1 | *Ucp1* | NM_009463.3 | aagctgtgcgatgtccatgt  aagccacaaaccctttgaaaa |

**Supplemental References**

1. Mina, A.I., et al., *CalR: A Web-Based Analysis Tool for Indirect Calorimetry Experiments.* Cell Metab, 2018. **28**(4): p. 656-666 e1.
